# The N-Terminal Part of *Drosophila* CP190 Is a Platform for Interaction with Multiple Architectural Proteins

**DOI:** 10.3390/ijms242115917

**Published:** 2023-11-02

**Authors:** Anton Golovnin, Larisa Melnikova, Valentin Babosha, Galina V. Pokholkova, Ivan Slovohotov, Anastasia Umnova, Oksana Maksimenko, Igor F. Zhimulev, Pavel Georgiev

**Affiliations:** 1 Department of Drosophila Molecular Genetics, Institute of Gene Biology, Russian Academy of Sciences, 34/5 Vavilov Street, Moscow 119334, Russia; 2 Department of the Control of Genetic Processes, Institute of Gene Biology Russian Academy of Sciences, 34/5 Vavilov Street, Moscow 119334, Russia; 3 Center for Precision Genome Editing and Genetic Technologies for Biomedicine, Institute of Gene Biology, Russian Academy of Sciences, 34/5 Vavilov Street, Moscow 119334, Russia; 4 Laboratory of Molecular Cytogenetics, Institute of Molecular and Cellular Biology of the Siberian Branch of the Russian Academy of Science, Novosibirsk 630090, Russiazhimulev@mcb.nsc.ru (I.F.Z.)

**Keywords:** architectural C2H2 proteins, CP190, Su(Hw), Chromator, Z4, M1BP

## Abstract

CP190 is a co-factor in many *Drosophila* architectural proteins, being involved in the formation of active promoters and insulators. CP190 contains the N-terminal BTB/POZ (Broad-Complex, Tramtrack and Bric a brac/POxvirus and Zinc finger) domain and adjacent conserved regions involved in protein interactions. Here, we examined the functional roles of these domains of CP190 in vivo. The best-characterized architectural proteins with insulator functions, Pita, Su(Hw), and dCTCF, interacted predominantly with the BTB domain of CP190. Due to the difficulty of mutating the BTB domain, we obtained a transgenic line expressing a chimeric CP190 with the BTB domain of the human protein Kaiso. Another group of architectural proteins, M1BP, Opbp, and ZIPIC, interacted with one or both of the highly conserved regions in the N-terminal part of CP190. Transgenic lines of *D. melanogaster* expressing CP190 mutants with a deletion of each of these domains were obtained. The results showed that these mutant proteins only partially compensated for the functions of CP190, weakly binding to selective chromatin sites. Further analysis confirmed the essential role of these domains in recruitment to regulatory regions associated with architectural proteins. We also found that the N-terminal of CP190 was sufficient for recruiting Z4 and Chromator proteins and successfully achieving chromatin opening. Taken together, our results and the results of previous studies showed that the N-terminal region of CP190 is a platform for simultaneous interaction with various DNA-binding architectural proteins and transcription complexes.

## 1. Introduction

In higher eukaryotes, the regulation of gene expression is a complex process which ultimately leads to cell differentiation during embryonic development. Cell specialization is determined by differences in transcription factor (TF) repertoires, and the genes responsible for cell differentiation and development of organisms are typically regulated by multiple independent enhancers, each of which stimulates a promoter in a limited population of cells during a specific time interval [1,2,3,4,5,6,7,8,9]. In contrast, housekeeping genes usually have enhancers located in close proximity to regulated promoters [10,11]. Special elements called chromatin boundaries or insulators are thought to be responsible for restricting the interactions between enhancers and promoters [12,13,14,15,16,17,18].

A number of studies have shown that the promoter can act as an insulator in some cases [13,19,20,21], suggesting the presence of common proteins with different functions involved in these regulatory elements. CP190, the 190 kDa centrosome-associated protein, is one such protein that binds predominantly to the promoters of housekeeping genes and insulator/boundary elements [22,23]. CP190 was initially identified within a cytoplasmic scaffolding complex that includes the centrosomal proteins [24]. Further study showed that during mitosis, CP190 is localized at the centrosomes and the mitotic spindle, but during interphase, CP190 binds to chromatin in the nucleus [25,26,27]. CP190 (1096 aa) contains an N-terminal Broad-complex, Tramtrack, and Bric-à-brac (BTB) domain and a cluster consisting of four C2H2-type zinc-finger domains (C2H2 domains) located in the central part of the protein [28,29]. According to several crystallization studies, the BTB domain of CP190 forms stable homodimers, and it has a high level of similarity to the BTB domains of mammalian proteins such as Kaiso, Bcl6, and PLZF that also contain clusters of C2H2 domains involved in DNA binding [25,30,31,32,33,34,35,36].

The *Drosophila melanogaster* genome contains only four genes encoding C2H2 proteins that have BTB-forming stable homodimers [33]. In contrast to mammalian BTB-C2H2 proteins, the four C2H2 domains of CP190 appear to be involved in protein–protein interactions rather than in DNA binding [28].

It has been shown that CP190 is recruited to regulatory elements via interactions with DNA-binding transcription factors [37,38,39,40,41]. The CP190 BTB resembles the previously characterized human BCL6 BTB domain that uses its hydrophobic groove to specifically associate with unstructured regions of several transcriptional repressors [42,43,44]. The CP190 BTB domain interacts similarly with conserved regions in the Pita, Su(Hw), CG31365, CG4730, and dCTCF proteins [31,45,46,47]. In addition, CP190 has at least two conserved regions between the BTB and the C2H2 cluster that interact with many architectural proteins, including Zipic, Opbp, CG31365, CG4730, and dCTCF [31,39,45,48].

CP190 predominantly associates with gene promoters and is required to support the opening of the chromatin [23,49,50,51]. ZIPIC, M1BP, and Opbp bind to their motifs in the promoters of housekeeping genes and are involved in recruiting CP190 [39,48,50]. Several studies have suggested that CP190 is responsible for the association of the NURF, dREAM, SAGA, and other complexes with regulatory elements [52,53,54,55]. In the Bithorax complex, the CP190 protein binds to all boundaries to ensure independent activity of transcriptional domains [23,56,57,58]. Each of the domains activates one of three homeotic genes, *Ubx*, *abd-A*, or *Abd-B*, in the appropriate parasegment/segment during *Drosophila* development [59,60,61,62]. Many boundaries have different combinations of binding sites for Pita, Su(Hw), and dCTCF that function in concert [63]. It has been demonstrated that CP190 is required for boundary and chromatin-opening functions [45,50,64,65]. Since Pita or dCTCF mutants lacking CP190-interacting domains are functional [45,46], it seems likely that CP190 is recruited to regulatory elements through interaction with several DNA-binding proteins.

The aim of this study is to finely map the CP190 domains that are responsible for interaction with various C2H2 proteins and to examine the functional role of these CP190 domains in vivo. We mapped two CP190 regions that interact with the ZIPIC, M1BP, and Opbp proteins. ZIPIC and Opbp interact with both the 210–245 aa and 309–390 aa regions, while M1BP interacts only with the 210–245 aa region. The deletion of these regions strongly affected CP190 binding to the chromatin. The BTBs of CP190 and human Kaiso showed structural similarity but low sequence homology. Kaiso BTB was able to partially substitute for the functions of the BTB domain in CP190, but it could not effectively recruit the CP190^hk^ chimeric protein to regulatory elements associated with dCTCF and Su(Hw) proteins. We also found that the N-terminal region of CP190 is sufficient to open chromatin and recruit the partner proteins Chromator (Chro/Chriz) and Z4 (Putzig/Pzg).

## 2. Results

### 2.1. Mapping the Domains of CP190 Required for Interaction with Architectural Proteins

**Figure 1:**
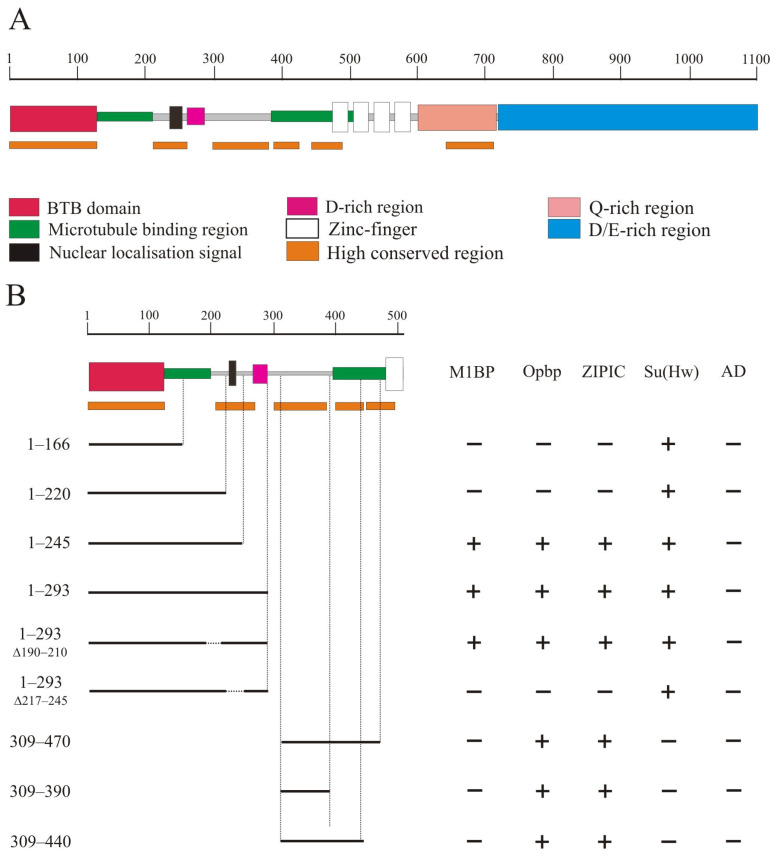
Precise mapping of two conserved N-terminal regions of CP190 that interact with architectural proteins. (**A**) Schematic presentation of the CP190 domain structure. The scale rule (in aa) is shown at the top of the figure. The highly conserved regions for the CP190 protein among *Drosophila* species are marked with orange lines. (**B**) Mapping of CP190 domains interacting with Opbp, M1BP, and ZIPIC proteins in the Y2H assay. Variants of CP190 were fused to the GAL4 DNA-binding domain and tested for interaction with the C2H2 proteins fused to the GAL4 activating domain. The results are summarized in columns with “+” and “−” signs referring to the presence and absence of interaction, respectively. The growth assay yeast plates are shown in Supplementary Materials Figure S3.

The CP190 protein (Figure 1A) contains the BTB domain (1–126 aa), a nuclear localization signal NLS (207–263), a D-rich domain (262–286 aa), four zinc-finger domains of the C2H2 type (471–594 aa), a Q-rich domain (647–717 aa), and a D/E-rich domain (727–1096 aa) [25,28]. In addition, 126–209 aa and 385–508 aa regions of CP190 are responsible for microtubule binding during mitosis [25,26].

It was previously shown that CP190 interacts with a large group of architectural C2H2 proteins. Five C2H2 proteins, including the well-known insulator proteins Su(Hw), dCTCF, and Pita, primarily interact with the BTB domain [31,47]. Three other C2H2 proteins, M1BP, Opbp, and ZIPIC, that bind to the promoters of housekeeping genes, interact with other regions of CP190 but not with the BTB domain. In a previous study [39], it was shown that ZIPIC interacts with the 245–468 aa region of CP190. Opbp was shown to interact with two overlapping regions in CP190: 1–309 aa and 245–468 aa [48]. The domain in CP190 responsible for interaction with M1BP has not yet been mapped [40].

We used a yeast two-hybrid assay to precisely map the domains in CP190 involved in the interaction with M1BP, Opbp, and ZIPIC (Figure 1B). A series of the N-terminal CP190 fragments fused with the GAL4 DNA-binding domain were tested for interaction with the C2H2 proteins fused with the GAL4 activation domain. The results showed that Opbp and ZIPIC interacted with the two non-overlapping CP190 regions located between the 1–245 aa and 309–470 aa regions. M1BP only interacted with the 1–245 region. Next, we mapped the interacting regions of CP190 more precisely to the 210–245 aa and 309–390 aa regions. Opbp and ZIPIC interacted with both CP190 regions simultaneously, while M1BP only interacted with the 210-245 aa region. Interestingly, the identified regions are preserved not only in Drosophilidae (Supplementary Figure S1) but also in Diptera (Supplementary Figure S2). The 210–245 aa and 309–390 aa regions are located in close proximity but do not overlap the CP190 regions that are involved in microtubule binding (Figure 1A).

### 2.2. Testing the Functional Role of CP190 Domains Required for Interaction with the Architectural C2H2 Proteins

**Figure 2:**
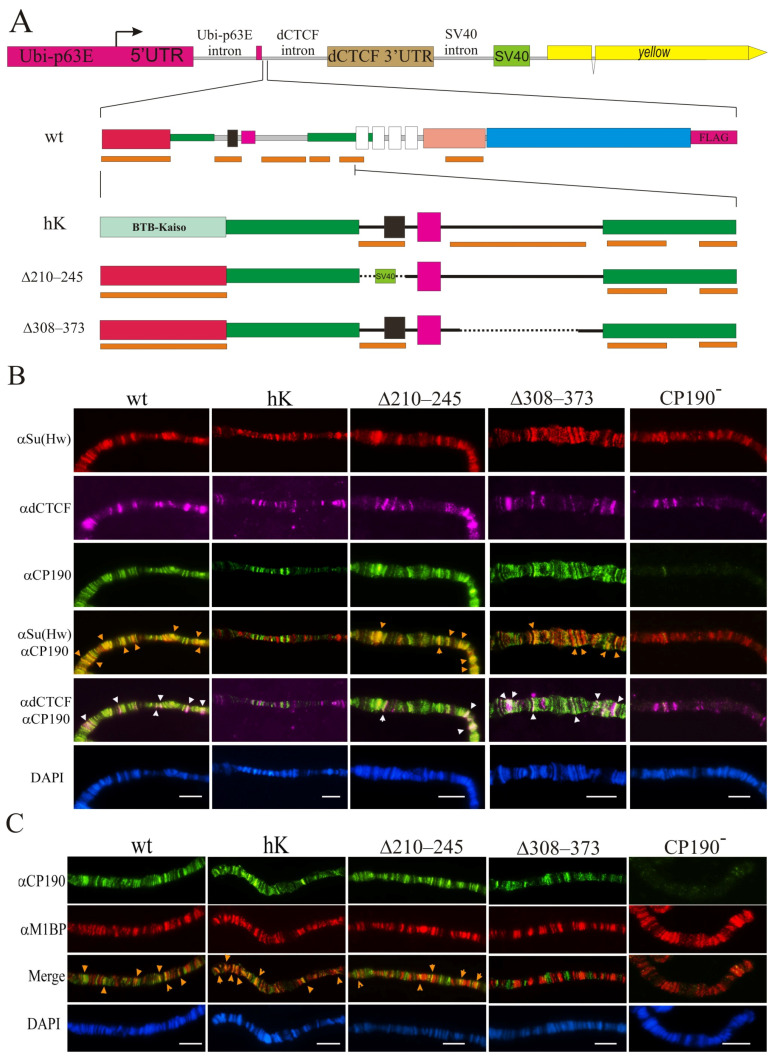
Binding of the CP190 variants to polytene chromosomes. (**A**) Schematic representation of the CP190 variants. The expression vector is shown above: the *Ubi-p63E* promoter with its 5′UTR and 3′UTR from the *dCTCF* gene fused with the SV40 region including polyadenylation signals. The intronless *yellow* gene (not to scale) was used as a reporter. Below are shown the full-length schemes of the CP190^WT^-F coding region and the N-terminal regions of the CP190^hK^–F, CP190^Δ210–245^–F, and CP190^Δ309–390^–F derivatives. Other designations are as in Figure 1. Cytological localization (**B**) of CP190, dCTCF, and Su(Hw) and (**C**) CP190 and M1BP proteins on the polytene chromosomes of the Ubi:CP190*/CyO; *Cp190^2^/Cp190^3^* lines, where Ubi:CP190* is either *Ubi:CP190^WT^*(WT), *Ubi:CP190^hK^*(hK), *Ubi: CP190^Δ210–245^*(Δ210–245), or *Ubi:CP190^Δ309–373^*(Δ309–373). CP190^−^ represents the *Cp190^2^/Cp190^3^* mutant line. The panels show the immunostaining using (**B**) anti-Su(Hw) (red), mouse anti-dCTCF (magenta), and rat anti-CP190 (green) antibodies, and (**C**) the rat anti-CP190 (green) and rabbit anti-M1BP (red) antibodies. Arrows indicate sites of protein colocalization. DAPI staining of polytene chromosome squashes is represented in blue. Scale bars, 10 μm.

Our previous results [31,39,45,46] showed that the BTB domain is required for the interaction of CP190 with a large group of C2H2 proteins. The BTB domain is critical for the stability and functions of CP190 [28], and thus most mutations in this domain destabilize its structure. The CP190 BTB domain [25,30,31] displays high sequence homology to the BTB domains of human Kaiso mammalian transcription factor clusters of the C2H2 domains (Supplementary Figure S4). The BTB domain of the human Kaiso protein had 16% amino acids identity, and 30% and 21% strong and weak amino acids similarity with the CP190 BTB domain [66,67], respectively. At the same time, the structural homology among *Drosophila* BTB CP190 and the BTB of the human Kaiso protein appeared incomplete (Supplementary Figure S4). In the Y2H assay, the Kaiso BTB domain did not interact with dCTCF, Su(Hw), or Pita, in contrast to the CP190 BTB domain (Supplementary Figure S5). To examine whether the interaction of BTB with C2H2 proteins was important for CP190 recruitment to chromatin, we obtained a transgenic line that expressed the chimeric CP190^hK^ protein in which the BTB domain was taken from the human Kaiso protein. To express the chimeric CP190^hK^ targeted by the FLAG epitope at the C-terminus of the fly protein, we inserted the transgene encoding CP190^hK^–F under the control of the strong ubiquitous ubiquitin (Ubi) promoter into the 38D region using the φC31-based integration system [68] (Figure 2A).

We also examined the functional role of the 210–245 aa and 309–390 aa regions of CP190 responsible for the interaction with the M1BP, Opbp, and ZIPIC proteins. We used the same expression vector and obtained CP190^Δ210–245^–F, CP190^Δ308–373^–F, and a control CP190^WT^–F driven by the Ubi promoter inserted in the same 38D genomic region (Figure 2A). It was shown previously that the 210–245 aa region coincides with the nuclear localization signal [25]. For this reason, we substituted the 210–245 aa with the NLS from SV40 (CP190^Δ210–245^–F). In the 309–390 region, we deleted the conserved 308–373 aa in CP190^Δ308–373^–F. Western blots showed that CP190^Δ210–245^–F, CP190^Δ309–390^–F, and CP190^hK^–F were expressed at similar levels as the control CP190^WT^–F, suggesting that all CP190 variants displayed normal stability (Supplementary Figure S6).

To examine the functional activity of the CP190 variants, we crossed the transgenes into the *Cp190*^−^ background. We used previously characterized null mutations in the *Cp190* gene: *Cp190^2^* and *Cp190^3^* [26]. The *Cp190^2^*/*Cp190^3^* trans heterozygotes died during the late embryonic stages, and less than 10% developed into pharate adults. Thus, the maternal effect of wild-type CP190 protein, inherited from wild-type parents, allows a small proportion of the *Cp190* null mutants to survive to pharate adults. The *Ubi:CP190^WT^* transgene completely restored the viability of *Cp190^2^*/*Cp190^3^*, even in the heterozygous state (Supplementary Figure S6). The *Ubi:CP190^hK^*/*CyO* partially restored the viability of *Cp190^2^*/*Cp190^3^*: approximately 3% (2.6%) survived to three-day-old adult flies that showed a normal phenotype and remained fertile. The homozygotes *Ubi:CP190^hK^*/*Ubi:CP190^hK^*; *Cp190^2^*/*Cp190^3^* survived longer: about 15% of the offspring reached the adult stage. The flies were viable and fertile, but their progeny died at the embryonic and first instar larval stages. These results suggest that CP190^hK^ protein expression cannot compensate for the functions of the wild-type protein. Moreover, the *Ubi:CP190^hK^*/*Ubi:CP190^hK^*; *Cp190^2^*/*TM6,Tb*, and *Ubi:CP190^hK^*/*Ubi:CP190^hK^*; *Cp190^3^*/*TM6,Tb* lines could not be maintained for several generations due to low viability and fertility. Thus, a high concentration of CP190^hK^ negatively affects the activity of the wild type CP190 protein, the amount of which is lower in a heterozygote for the *Cp190* null mutation.

Similar to *Ubi:CP190^hK^*, both *Ubi:CP190^Δ210–245^* and *Ubi:CP190^Δ308–373^* in the heterozygous state were insufficient to complement the *Cp190^2^*/*Cp190^3^* mutations; the adult flies died within days of hatching (Supplementary Figure S7). The *Cp190^−^* flies homozygous for the *Ubi:CP190^Δ308–373^* or *Ubi:CP190^Δ210–245^* transgenes that developed to the adult stage were less viable than *Ubi:CP190^hK^* (less than 7%), and they died soon after hatching. Also, strong expression of Ubi:CP190^Δ210–245^ or Ubi:CP190^Δ308–373^ negatively affected the viability of flies heterozygous for the *Cp190^2^* or *Cp190^3^* mutations. Thus, each of the mutant proteins Ubi:CP190^hK^, Ubi:CP190^Δ210–245^, and Ubi:CP190^Δ308–373^ displayed similar effects on *Drosophila* development.

### 2.3. The Ability of CP190 Variants to Bind to Regulatory Elements

Recent studies have shown that the interbands of polytene chromosomes typically correspond to the promoter regions of broadly expressed housekeeping genes and display an “open” chromatin conformation [69]. CP190 binds to many interband regions on the polytene chromosomes of third-instar larvae and extensively co-localizes with dCTCF, Su(Hw), PITA, ZIPIC, and M1BP proteins [23,39,50,70].

To determine whether the mutant variants of CP190 would bind to identical genomic sites and co-localize with Su(Hw), dCTCF, and M1BP the same as the wild-type proteins, we analyzed the binding patterns of the CP190 variants expressed from one copy of the transgene (Ubi:CP190*/*CyO*) in *Cp190^2^/Cp190^3^* larvae. The degree of co-localization of CP190 variants with Su(Hw) or dCTCF (insulator and promoter sites) and M1BP (mainly housekeeping promoters) on the polytene chromosomes of third instar larvae was studied (Figure 2B,C).

CP190^WT^ bound to hundreds of interbands and extensively co-localized with Su(Hw), dCTCF, and M1BP on the polytene chromosome of *Ubi:CP190^WT^*/*CyO*; *Cp190^2^*/*Cp190^3^* larvae (Figure 2B,C; Supplementary Figures S8 and S9). In *Ubi:CP190^hK^*/*CyO*; *Cp190^2^/Cp190^3^* larvae, the total number of CP190^hK^-labeled sites was reduced, and co-localization with Su(Hw) and dCTCF sites was considerably lower than with CP190^WT^ (Figure 2B, Supplementary Figure S8). At the same time, we observed strong co-localization between CP190^hK^ and M1BP, similar to CP190^WT^ (Figure 2C; Supplementary Figure S9).

Comparison of polytene chromosomes in larvae expressing CP190^WT^ or CP190^Δ210–245^ suggested that the CP190^∆210–245^ protein bound as efficiently as CP190^WT^ (Figure 2B,C, Supplementary Figures S7 and S8). CP190^Δ308–373^ bound to the polytene chromosome less efficiently than CP190^Δ210–245^ but was still present at most bands enriched by dCTCF and Su(Hw) (Figure 2B; Supplementary Figure S8). Thus, unlike CP190^hK^, CP190^Δ308−373^ and CP190^Δ210–245^ continued to bind to the dCTCF and Su(Hw) sites. At the same time, co-localization of CP190^Δ308–373^ and M1BP was significantly reduced (Figure 2C and Figure S8).

Overall, our results suggest that the binding of CP190^hK^ to dCTCF/Su(Hw) sites was reduced, and that this was correlated with the main role of the BTB domain of CP190 in interaction with dCTCF and Su(Hw). At the same time, CP190^Δ308–373^ and CP190^Δ210–245^ bound more efficiently to the dCTCF and Su(Hw) sites and co-localized less efficiently with M1BP.

**Figure 3:**
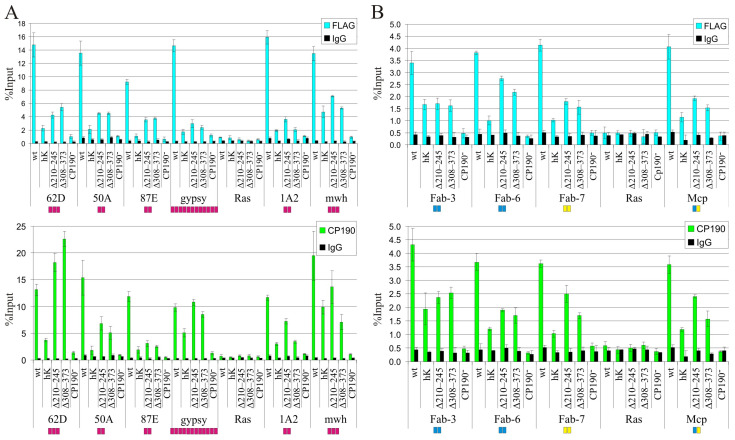
The results of experiments designed to evaluate the ability of the CP190 variants to bind the selected chromatin sites. ChIP-qPCR analysis of CP190 binding to chromatin was performed using third-instar larvae of transgenic lines expressing different variants of the CP190 protein: CP190^WT^-F (wt), CP190^hK^–F (hK), CP190^Δ210–245^–F (Δ210–245), CP190^Δ309–373^–F (Δ309–373), and control *Cp190^2^/Cp190^3^* (Cp190^−^). The most well-characterized CP190-bound sites associated with different architectural proteins were studied: (**A**) Su(Hw) binding regions. Magenta squares indicate the number of Su(Hw) binding sites in the tested regions. (**B**) Boundaries in the Bithorax complex. Blue squares indicate the number of dCTCF binding sites, and yellow squares indicate the number of Pita binding sites in the tested regions. Immunoprecipitation was performed with either FLAG (blue bars) or CP190 (green bars) antibodies. PCR products were amplified from three separate immunoprecipitates of three different chromatin preparations. The *ras64B* coding region (Ras) was used as a control, being devoid of CP190 binding regions. The percentage recovery of immunoprecipitated DNA (Y axis) was calculated relative to the amount of input DNA. Error bars indicate standard deviations of three independent biological replicates.

To further analyze the specificity of recruiting by CP190 variants, we compared the binding of CP190 variants by qChIP to the main well-described binding sites for the Su(Hw) protein (Figure 3A). The 50A, 62D, and 87E sites are intergenic regions containing 2-3 Su(Hw) binding sites [71]. The 1A2 region is located downstream from the 3′ side of the *yellow* gene and contains two Su(Hw) binding sites [72]. The *gypsy* insulator consists of the 12 binding sites for Su(Hw) [73,74]. For the qChIP analysis, we used chromatin obtained from *Cp190^2^/Cp190^3^* larvae heterozygous for either of the CP190 transgenes. For all tested sites, CP190^WT^ bound more efficiently than the mutant versions of CP190 (Figure 3A). In accordance with the results obtained for the polytene chromosomes, CP190^Δ210–245^ bound to the Su(Hw) sites with similar or better efficiency than CP190^Δ308–373^. Finally, CP190^hK^ bound with the same or weaker efficiency than CP190^Δ308–373^. Thus, the results were consistent with the hypothesis that the BTB domain of CP190 is critical for binding to Su(Hw) sites [31,47].

We also tested the binding of CP190 variants to the well-described boundaries in the Bithorax complex (BX-C) (Figure 3B). The selected study boundaries contain different combinations of dCTCF and Pita sites: Mcp (Pita + dCTCF), Fab-6 (2 dCTCF), Fab-7 (2 Pita), and Fab-3 (2 dCTCF) [75]. The qChIP analysis showed that the patterns of binding efficiency of CP190 variants are preserved for the BX-C boundaries: the most efficient binding is observed for CP190^Δ210–245^, and less efficient for CP190^hK^ (Figure 3B).

### 2.4. Identification of the CP190 Domain Required for the Formation of the Interband Region in Larval Polytene Chromosomes

**Figure 4:**
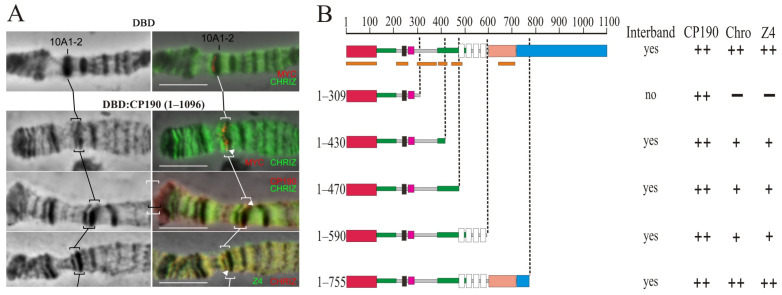
Mapping of the region in CP190 required to organize interbands in a polytene chromosome model. (**A**) Targeting the GAL4 DNA-binding region fused with the *myc* epitope (DBD) and the 1–1096 aa CP190 regions fused with the GAL4 DNA-binding region and the *myc* epitope (DBD:CP190 1–1096) to the 16 GAL4 binding sites in the 10A1-2 disc. The left panel demonstrates the polytene chromosomes in phase contrast. The right panel is an overlay of phase contrast and immunostaining with antibodies against *myc* (red), Chriz (green), Z4 (green), and CP190 (red). Black and white square brackets and white arrows indicate the model 10A1-2 bands in different polytene chromosome preparations. At the top, the recruitment of the GAL4 DNA-binding region alone (DBD) did not induce the formation of the interband in the 10A1-2 band (negative control). At the bottom, the recruitment of the GAL4 DNA-binding region fused with 1–1096 aa CP190 induces the formation of the interband in the 10A1-2 band (positive control). Scale bars, 10 μm. (**B**) Summary of the results for attracting truncated versions of CP190 to 16xGAL4 binding sites in the 10A1-2 band. The truncated versions of CP190 were fused with the GAL4 DNA-binding region: DBD:CP190(1–309), DBD:CP190(1–430), DBD:CP190(1–470), DBD:CP190(1–590), and DBD:CP190(1–755). “Interband” represents the presence (“yes”) or absence (“no”) of the interband in the 10A1-2 band model system. The results of CP190, Z4, and Chriz protein recruitment are summarized in columns with “++”, “+” and “−” signs referring to the full, partial presence, or absence, respectively, of tested proteins in the generated interband in the 10A1-2 band. Other terms are as in Figure 1.

The CP190 protein binds to promoters of housekeeping genes and is involved in the organization of the opened chromatin [23,50]. Interbands have been reported to be preferentially associated with the CP190, Chromator (Chro/Chriz), and Z4 (Putzig/Pzg) proteins [76]. To map the CP190 domains involved in chromatin opening, a model system was used [77] in which 14 GAL4 binding sites were inserted into the 10A1-2 region, which in the polytene chromosomes of larvae is an inactive chromatin band. The CP190 protein and its truncated derivatives were fused in-frame with the DNA-binding domain of the yeast GAL4 (DBD) under the control of the hsp70 promoter. The transgenes expressing the chimeric proteins were inserted into the 51C region on the second chromosome using the φC31-based integration system [68]. A series of transgenic lines were obtained, each of which had the 10A1-2 insert combined with one of the DBD:CP190 variants (Figure 4). To express the chimeric protein, the flies were maintained at 29 °C from the embryonic to the pupal stages. As a negative control, a transgenic line expressing only the GAL4 binding region (DBD) was used [77]. In this transgenic line, the DBD is recruited to the 10A1-2 region but does not change the polytene organization and fails to recruit other proteins (Figure 4A).

The expression of the complete CP190 (DBD:CP190^WT^) gave rise to a prominently decondensed zone and formation of the interband that splits the 10A1-2 band (Figure 4A,B). On the polytene chromosomes, CP190 actively bound to the decondensed region of the interband, which also contains Chromator and Z4 proteins. Thus, the complete CP190 protein could recruit Z4 and Chromator proteins, suggesting their tight cooperation.

Expression of DBD:CP190(1–590), DBD:CP190(1–470), and DBD:CP190(1–430) induced the formation of interbands, but Z4 and Chro recruitment was less efficient (Figure 4B, Supplementary Figure S10A). It seems likely that the C-terminal (590–1097 aa) of CP190 contributes to the recruitment of Z4 and Chro. Finally, the expression of DBD:CP190 (1–309) did not induce interband formation or recruitment of Z4 or Chro (Figure 4B, Supplementary Figure S10B). Thus, the region of 309–470 aa, including two conserved regions (Supplementary Figures S1 and S2), may be responsible for both the interaction with the Z4 and Chro proteins and chromatin opening.

## 3. Discussion

The architectural C2H2 proteins employed different strategies for interaction with CP190. The dCTCF, Su(Hw), and Pita proteins interact predominantly with the BTB domain of CP190. These proteins bind to promoters and are responsible for the organization of the most well-characterized *Drosophila* insulators. The second group of architectural proteins, including M1BP, Opbp, and ZIPIC, interacted with one or two highly conserved regions located between 210–245 aa and 309–390 aa. The existence of several domains in CP190 involved in interactions with DNA-binding architectural proteins makes it possible to create an effective platform for in vivo recruitment of CP190 to regulatory elements. The regions involved in the interactions with architectural proteins are located close to the regions that are responsible for the association of CP190 with centrosomes. It is likely that such an organization of domains makes it possible to efficiently carry out the relocalization of CP190 from chromatin in the interphase to the centrosomes during mitosis.

The BTB domain of Kaiso has 67% sequence homology to the CP190 BTB domain [78,79]. Both domains form dimers, but the BTB domain of Kaiso cannot interact with the *Drosophila* architectural proteins. Our results showed that in the chimeric CP190^hK^, the Kaiso BTB could partially functionally replace the CP190 BTB domain. This suggests that the BTB domains may not only have a common ability for homodimerization but may also interact with unknown protein complexes that are conserved among higher eukaryotes. Thus, the CP190^hk^ chimeric protein can be used to identify conserved transcriptional complexes among higher eukaryotes that are recruited to the Kaiso BTB domain. Mammalian BTB-C2H2 proteins, including Kaiso, are important transcriptional regulators involved in organismal development and cell differentiation [32,33,80,81]. Impaired expression of BTB-C2H2 proteins is closely correlated with tumorigenesis [82,83,84,85,86,87,88,89]. Currently, anticancer inhibitors are being actively developed that bind to individual regions of the BTB domain and block interactions with partner proteins [90,91,92,93,94,95,96,97]. It seems likely that chimeric Drosophila proteins carrying the BTB domains of key mammalian transcription factors can be successfully used to identify and test new anticancer drugs.

Flies expressing chimeric CP190^hK^, CP190^Δ210–245^, or CP190^Δ308–373^ displayed similar phenotypes: strong reduction in viability and fertility of adults, and high levels of larval and pupal mortality. Thus, all three regions of CP190 may have similar essential functional roles as well as being highly conserved in different Dipteran species. In contrast, the 126-209 aa microtubule binding domain, the C2H2 domains, and the C-terminal D/E rich region are not essential for CP190 functions in vivo [25,28]. Thus, the CP190 protein consists of several structural modules that perform different functions.

On the polytene chromosomes, it was possible to study the efficiency of CP190 binding to the promoters of active genes and insulators located in the interbands. Only the most efficient CP190 binding sites are visible on polytene chromosomes, making it possible to assess the efficiency of CP190 variants binding to different architectural proteins. We obtained the clear result that CP190^hK^ binds less effectively to the regions that are associated with the dCTCF and Su(Hw) proteins. This result supported the hypothesis of a key role for the BTB domain in the interaction of CP190 with this group of architectural C2H2 proteins.

M1BP along with ZIPIC and Opbp form another group of architectural C2H2 proteins that predominantly bind to housekeeping promoters and interact with the CP190-Z4-Chromator complex. The 309–390 aa region is the most essential element for recruiting CP190 to polytene chromosomes. It is likely that the same or adjacent (located in 390–430 aa) region is required for interaction with Z4 and Chromator proteins. The Z4, Chromator, and CP190 proteins can form a platform on the housekeeping promoters for efficient recruitment of transcription and remodeling complexes such as NURF and dREAM [52,53,55] through multiple protein–protein interactions.

On the whole, the obtained results are consistent with the hypothesis that the CP190 protein constitutes a platform for interaction with various proteins and transcription complexes. The BTB domain and several conserved N-terminal regions are responsible for specific interactions of CP190 with architectural proteins and Chro/Z4 partner proteins. Different combinations of the architectural proteins determine the ability of CP190 and its partners to bind to the regulatory regions—predominantly promoters. The binding of CP190/Z4/Chro to regulatory elements results in the attraction of transcriptional complexes and, as a result, in switching on of regulatory elements. There are also a number of experimental data indicating that CP190/Z4/Chro can also participate in the organization of TADs and distance interactions between regulatory elements [51,98,99,100]. The CP190, Z4, and Chro proteins, by interacting with each other and homodimerizing, can support long-distance interactions between housekeeping gene clusters, which often form TAD boundaries [4]. Further studies are needed to understand the mechanisms of interaction between the CP190, Z4, and Chro proteins, as well as the degree of their interdependence in the recruitment of transcriptional complexes to regulatory elements and the maintenance of long-range interactions.

## 4. Materials and Methods

### 4.1. Plasmids and Cloning

#### 4.1.1. Plasmids for Y2H Assay

Plasmids for the yeast two-hybrid assay (Y2H) were prepared using the full-sized M1BP, Opbp, Zipic, and Su(Hw) fused with pGAD424 vector (Clontech, Mountain View, CA, USA).

To generate M1BP plasmid, 5′-tcg aattcATGTCGAAATCGGCGC-3′ (introduced site for EcoRIenzyme) and 5′-aggtcgacGATGTGCAGGCTGTC-3′ (introduced site for SalI enzyme) primers were used to amplify cDNA, followed by digesting with EcoRI and SalI enzymes (recognition sites underlined) and cloning in pGAD424 vector digested by the same enzymes.

To generate Opbp plasmid, 5′-ttggatcc aaATGTCATCGTCCAACCAGG-3′ (introduced site for BamHI enzyme) and 5′-ttgtcgacGTTATTACAGATTGGCTTAAG-3′ (introduced site for SalI enzyme) primers were used to amplify cDNA, followed by digesting with BamHI and SalI enzymes (recognition sites underlined) and cloning in pGAD424 vector digested by the same enzymes.

To generate ZIPIC plasmid, 5′-ttg aattcATGAATTGCTGCATTTGTC-3′ (introduced site for EcoRI enzyme) and 5′-ttgtcgacAACTGGCGTGCAGTCGTC-3′ (introduced site for SalI enzyme) primers were used to amplify cDNA, followed by digesting with EcoRI and SalI enzymes (recognition sites underlined) and cloning in pGAD424 vector digested by the same enzymes.

To generate Su(Hw) plasmid, 5′-aggtcgacTCAAGCTTTCTCTTGTTC-3′ (introduced site for SalI enzyme (underlined)) and 5′-GTTAAACACATCAGCGGGCTC-3′ primers were used to amplify C part of Su(Hw) cDNA. Resulting PCR fragments were digested with EcoRI and SalI enzymes and cloned in pGAD424 vector digested by the same enzymes. Next, EcoRI-EcoRI fragment from Su(Hw) cDNA was cloned in obtained plasmid with orientation checking.

Truncated versions of CP190 were fused with pGBT9 vectors (Clontech, Mountain View, CA, USA).

To generate CP190 derivatives 1–166 aa, 1–220 aa, 1–245 aa, and 1–293 aa, one common 5′ cATGGGTGAAGTCAAGTCCGTG 3′ and set of various primers were used for each construct with introducing for BamHI (underlined):

166 rev 5′ tggatccttaTCGCGATTGCGGCGAAGG 3′

220 rev 5′ tggatccttaATAACCCTTTCGCAGCTGCTC 3′

245 rev 5′ tggatccttaCTTAACCTCTTCCAAACTGGG

293 rev 5′ tggatccttaTGTAGATGGCTGCGGTGGC 3′

Resulting PCR fragments were digested with BamHI and cloned in pGBT9 or pGAD424 vectors digested by SmaI and BamHI.

To generate 1–293 CP190 derivatives with either 190–210 aa or 217–242 aa deletions, we used in-fusion cloning mutagenesis technology [101]. PCR-fusion involves two parallel PCR amplifications from pGBT9 CP190_1–293 template with:

rev190 5′ CTCGAATGGTGACGTTGTTCGGCTCATTGTG 3′,

dir210 5′ ACAATGAGCCGAACAAGTCACCATTCGAGC 3′

primers for 190–210 aa deletion; and:

rev217 5′ TTCCTTAACCTCTTCTCGCAGCTGCTCGAAT 3′,

dir242 5′ TTCGAGCAGCTGCGAGAAGAGGTTAAGGAAT 3′

primers for 217–242 aa deletion. PCR fusion of the amplified fragments through a single overlap extension was carried out on gel-purified PCR fragments from the parallel reactions.

To generate CP190 derivatives 309–470 aa, 309–390 aa, and 309–440 aa one common 5′ATCCACCACCATTATCTTGAAACAG 3′ and set of various primers were used for each construct with introducing site for BamHI (underlined):

470 rev 5′ tggatccttaCCAGGGCCCAGTAGTATTC 3′

390 rev 5′ tggatccttaAGTTGCGGATGACTGGTTCG 3′

440 rev 5′ tggatccttaTGCTGGTGGATTCGCCC 3′

Resulting PCR fragments were digested with BamHI and cloned in pGBT9 vector digested by SmaI and BamHI.

To generate CP190 BTB, we digested pGBT9 CP190_1–166 or pGAD424 CP190_1–166 with BamHI and NcoI, then filled in with Klenow fragment and self-ligated plasmids.

To generate hKaiso BTB, we used primers:

hK-dir 5′ cATGGAGAGTAGAAAACTGATTTCTG 3′

hK-rev 5′ tggatccttaGCGCTGTACCTGAGATGC 3′

Resulting PCR fragments were digested with BamHI enzyme (underlined) and cloned in pGBT9 or pGAD424 vectors digested by SmaI and BamHI.

#### 4.1.2. Plasmids for Transgenic Lines Generation

Transgene line expressing full length CP190 protein was described previously [102].

To generate hKaiso-BTB CP190 derivative, hKaiso-BTB was amplified with primer

5′ ttactagtATGGAGAGTAGAAAACTGATTTCTG 3′, including recognition site for SpeI enzyme (underlined) and 5′ GCTGTACCTGAGATGCTTTTAAC 3′ primer. Resulting PCR product was digestated with SpeI and cloned in *pUbi-CP190-attB* vector digested by NcoI followed by fill in with Klenow fragment and SpeI.

To generate CP190 derivative with SV40 NLS positioned in 210–245 aa deletion, 5′ and 3′ sequences of SV40 NLS were introduced in 5′ ends of primers with recognition site for BsaI (SV40 sequences in capital letters, BsaI underlined):

CP190-NLS1-R 5′ ttggtctcaCTTCTTGGGcagctgctcg aatggtgacgtt 3′

CP190-NLS2-F 5′ ttggtctcag aagAAGAGGAAGGTGgaggtt aagg aattcgctgag 3′.

Additional two primers with recognition site for NcoI and AgeI enzymes were used (recognition sites underlined):

CP190-NcoI-F 5′ gcaccgtcgcaccatggag aa 3′

CP190-AgeI-R 5′ ttccctggttaccggttgtc 3′

PCR products from CP190-NcoI-F/CP190-NLS1-R and CP190-AgeI-R/CP190-NLS2-F reactions were purified and digested with BsaI followed by ligation. Ligated product was used as matrix for second round of PCR with CP190-NcoI-F/CP190-AgeI-R primers. Resulting product was digested with NcoI and AgeI and ligated in *pUbi-CP190-attB* [102] vector digested by the same enzymes.

To generate CP190 derivative with 308–373 aa deletion, we digested PCR product generated by 5′ AAAATCTGCTCCAGCGACAGT 3′ and 440 rev 5′ tggatccttaTGCTGGTGGATTCGCCC 3′ primers by BstEII, and cloned it in *pUbi-CP190-attB* vector digested by BamHI followed by fill in with Klenow fragment and BstEII.

To generate CP190 derivative 1–1096 aa, 1–755 aa, 1–590 aa, 1–470 aa, 1–430 aa, and 1–309 aa tested in interband organization, we performed several PCRs with common 5′ atggcgcgccaATGGGTGAAGTCAAGTCC 3′ primer with introduced recognition site for AscI enzyme and different specific primers with introduced recognition site for NotI enzyme (recognition sites underlined):

1096_rev 5′ tgcggccgcttatagctcctccttcgccg 3′

755_rev 5′ aagcggccgcgTTCCAGGTTGTCAATGGAC 3′

590_rev 5′ aagcggccgcgCTTGTGGTAGCTCTTCATGTG 3′

470_rev 5′ aagcggccgcgCAGGGCCCAGTAGTATTC 3′

430_rev 5′ aagcggccgcgACCGGTTGTCTGCGAGGTT 3′

309_rev 5′ aagcggccgcgCATGTTCTAGTTGGGTCTGTG 3′

Resulting PCR products were digested with AscI and NotI and cloned in *hsp70*-GAL4DBD-MYC [77] digested with the same enzymes.

### 4.2. Germ-Line Transformation, Genetic Crosses, and Phenotypic Analysis

*Drosophila* strains were grown at 25 °C under standard culture conditions. The transgenic constructs were injected into preblastoderm embryos using the φC31-mediated site-specific integration system [68]. The emerging adults were crossed with the *y^1^w^1118^* flies, and the progeny carrying the transgene were identified by *y*^+^ pigmented cuticle. Details of the crosses are available upon request.

### 4.3. Yeast Two-Hybrid Assay

The Y2H assay was performed with plasmids and protocols from Clontech (Palo Alto, CA, USA). Yeast strain PJ69-4a (MATa trp1-901 leu2-3,112 ura3-52 his3-200 gal4(deleted) gal80(deleted) LYS2::GAL1-HIS3 GAL2-ADE2 met2::GAL7-lacZ) was used. Yeast clones from fresh YPD (2% Difco peptone, 1% yeast extract, 2% glucose, 1.5% agar) agar plate were inoculated into 0.5 mL YPD medium, incubated overnight with shaking at 30 °C and 250 r/min. Then, overnight culture was diluted ten times in new flask and grow in YPD medium until A600 0.4–0.6. Next, cells were precipitated 1000 g 5 min RT follow by supernatant discarding. Then, cells were resuspended in 25 mL of sterilized water, washed, and collected by centrifugation in the same conditions. Cells were resuspended in 1 mL fresh sterile 100 mM LiAC. These cells’ suspension was gently resuspended with 0.1 μg mix of BD/X and AD/Y plasmid previously prepared into each tube. Appropriate volume of 50% PEG 4000/100 mM LiAc was added following by gentle mix and incubation 30 °C, 200 r/min, with shaking for 0.5 h. Heat shock was performed for 15 min at 42C followed by 5 min on ice incubation. Then, cells were precipitated 2 min at 4000 g at room temperature. Resuspended in sterilized water and plated on first selective media (Yeast Nitrogen Base without Amino Acids (Sigma cat#Y0626, St. Louis, MO, USA), 2% glucose, 1.5 agar) with addition of all essential amino acids except tryptophan and leucine. After 3 days of growth at 30 °C, the cells were plated on the same selective media without tryptophan, leucine, histidine, and adenine, and their growth was compared after 2–3 days. Each assay was repeated three times.

### 4.4. Chromatin Preparation and ChIP Analysis

Chromatin was prepared from the third instar larvae stage, and the resulting chromatin preparation was used for ChIP experiments, as described previously [103]. Immunoprecipitated DNA was quantified using qPCR, with SYBR green (Bio-Rad Cat# 170-8882, Hercules, CA, USA). Primers were positioned in the middle of the binding region, as identified in ModEncode by ChIP-seq. The primer sequences used in PCR for ChIP analyses are shown in Supplementary Table S1. Analyses were performed on three biological replicates.

### 4.5. Antibodies

Antibodies to Su(Hw), CP190, dCTCF, Z4, and Chriz were generated and described previously [77,103,104]. Antibodies against M1BP (90 aa–240 aa) (provided by Anna Fedotova) were produced in rabbits and purified by affinity purification on Aminolink resin (ThermoFisherScientific, Waltham, MA, USA) according to standard protocols. Details are described in the Supplementary Materials.

### 4.6. Western Blot Analysis

Protein extracts were prepared by homogenization of 15 third-instar larvae or 25 flies in 100 μL of hot 60 mM Tris-HCl (pH = 6.8), 4% SDS, 10% glycerol, and 0.7 M β-mercaptoethanol, followed by boiling for 10 min. Debris was precipitated by centrifugation and 25 μL of supernatant was subjected to 7% polyacrylamide gel electrophoresis [105]. Proteins were electroblotted to PVDF membrane in 25 mM Tris-HCl (pH = 8.3), 192 mM glicine, and 20% methanol [106]. Membranes were blocked with 10% nonfat milk and incubated overnight with primary antibodies: anti-CP190 (rat) 1:5000, anti-FLAG (mouse) 1:5000, and anti-lamin Dm0 (clone ADL84.12, DSHB, Iowa City, IA, USA) 1:1000. Several washes with PBST buffer (1× PBS + 0.1% Teen20) were performed next day followed by incubation with secondary antibodies (HRP ant-Rat 1:10000 (Jackson_Immuno_Research, 112-035-175, West Grove, PA, USA) and HRP ant-mouse 1:10,000 (Jackson_Immuno_Research 115-035-174, West Grove, PA, USA)) for two hours. Then, several washes with PBST buffer were performed followed by developing with ECL kit (Thermo Scientific ECL Plus cat#32132, Rockford, IL, USA) and visualization with X-ray film.

### 4.7. Immunostaining of Polytene Chromosomes

*Drosophila* 3rd instar larvae were cultured at 18 °C under standard conditions. Polytene chromosome staining was performed as described [107]. The following primary antibodies were used: anti-CP190 (rat) 1:300, anti-Su(Hw) (rabbit) 1:300, anti-dCTCF 1:50 (mouse), anti-Chriz 1:600 (rabbit), anti-Z4 1:50 (mouse), and anti-M1BP (rabbit) 1:200. The applied secondary antibodies were Cy3-AffiniPure Donkey Anti-Rabbit 1:200 (Jackson Immuno Research 711-165-152, West Grove, PA, USA), Cy5-AffiniPure Donkey Anti-mouse 1:200 (Jackson Immuno Research 715-175-151, West Grove, PA, USA), and FITC-AffiniPure Donkey Anti-Rat 1:200 (Jackson Immuno Research 712-095-153, West Grove, PA, USA). The polytene chromosomes were co-stained with 4′,6-diamidino-2-phenylindole (DAPI) (AppliChem, Darmstadt, Germany). Analysis was performed with a Zeiss fluorescence microscope (Axio Observer.Z1, Jena, Germany), which has integrated an OptiGrid Structured Illumination Microscopy system (Qioptiq, Luxembourg). Fiji was used to process images: zooming in of selected regions and optimization brightness/contrasts of the images.

## Data Availability

All data generated or analyzed during this study are included in this published article and its Supplementary Materials files.

## Supplementary Materials

The following supporting information can be downloaded at: https://www.mdpi.com/article/10.3390/ijms242115917/s1.
