# Supplementary material for: The N-Terminal Part of Drosophila CP190 Is a Platform for Interaction with Multiple Architectural Proteins
Source: Int J Mol Sci. 2023 Nov 2;24(21):15917. doi: 10.3390/ijms242115917 (PMC10648081; doi:10.3390/ijms242115917)
Supplement: Supplementary file 1 [file ijms-24-15917-s001.zip › ijms-2673324-supplementary.pdf]

Supplementary information for

The N-terminal part of *Drosophila* CP190 is a platform for interaction with multiple architectural proteins

Anton Golovnin[1,*], Larisa Melnikova[1], Valentin Babosha[2,3], Galina V. Pokholkova[4], Ivan Slovohotov[3], Anastasia Umnova[2], Oksana Maksimenko[3], Igor F. Zhimulev[4], Pavel Georgiev[2,*]

[1]Department of Drosophila Molecular Genetics, Institute of Gene Biology, Russian Academy of Sciences, 34/5 Vavilov St., 119334, Moscow, Russia; agolovnin@mail.ru (A.G); lsm73@mail.ru (L.M.)
[2]Department of the Control of Genetic Processes, Institute of Gene Biology Russian Academy of Sciences, 34/5 Vavilov St., Moscow 119334, Russia; v.babosha@gmail.com (V.B.); anast.popovich@gmail.com (A.U.); georgiev_p@mail.ru (P.G.)
[3]Center for Precision Genome Editing and Genetic Technologies for Biomedicine, Institute of Gene Biology, Russian Academy of Sciences, 34/5 Vavilov St., Moscow 119334, Russia; v.babosha@gmail.com (V.B.); ivan_slovohotov@mail.ru (I.S.); maksog@mail.ru (O.M.)
[4]Laboratory of Molecular Cytogenetics, Institute of Molecular and Cellular Biology SB RAS, 630090 Novosibirsk, Russia; galina@mcb.nsc.ru (G.V.P.); zhimulev@mcb.nsc.ru (I.F.Z.)

*Corresponding authors:

E-mail: georgiev_p@mail.ru (PG)

E-mail: agolovnin@mail.ru (AG)

Table S1. Primer sequences used in ChIP-qPCR analysis

Primer pair	Sequences
62D-Fw<br>62D-Rev	5’ TTTGGGCTTGGTGAGAACAG 3’<br>5’ TGATACCAGGCGAACAGAAATC 3’
50A-Fw<br>50A-Rev	5’ ATACAAAGTGGTTTCAGCCAAGAAG 3’<br>5’ TTGATAAATAGTCCAGCACGCATAC 3’
87E-Fw<br>87E-Rev	5’ GGATGTTACATTGAGAGTGCTTAGG 3’<br>5’ TTTGCGTTTCGGCTGCTGTC 3’
gypsy-Fw<br>gypsy-Rev	5’ TTCTCTAAAAAGTATGCAGCACTT 3’<br>5’ CACGTAATAAGTGTGCGTTGA 3’
1A2-Fw<br>1A2-Rev	5’ ACCACACATCAGTCATCGTGT 3’<br>5’ CTTCGTCTACCGTTGTGC 3’
Ras-Fw<br>Ras-Rev	5’ GAGGGATTCCTGCTCGTCTTCG 3’<br>5’ GTCGCACTTGTTACCCACCATC 3’
mwh-Fw<br>mwh-Rev	5' -CGATGGTTGACAGGTGAGCAA- 3'<br>5' -TGGTTGCTAAGAACTTCGTTCG- 3'
Fab3_cts_d<br>Fab3_cts_r	5' TAAAGGCCAATGCACAAAGGCGAC 3’<br>5' ACGCTTCAGCGAACGGAATACAGA 3’
MCP_cts_d<br>MCP_cts_r	5’AAAGTCGGGTCTGCAAATAAGG 3’<br>5’ GCATAAGCTGCAAAAGAAAAACAA 3’
Fab6_cts_d<br>Fab6_cts_r	5’ AGCTAAACCCGATTTGCTTTGCCG 3’<br>5’ CTGCCCAGTGGGAGATACAAAGAT 3’
F7-RTd<br>F7-RTr	5’ TAAGCCAACTGGTTTCCAACTCT 3’<br>5’ TTGCCCAGGGTAAGTAACGGTAT 3’

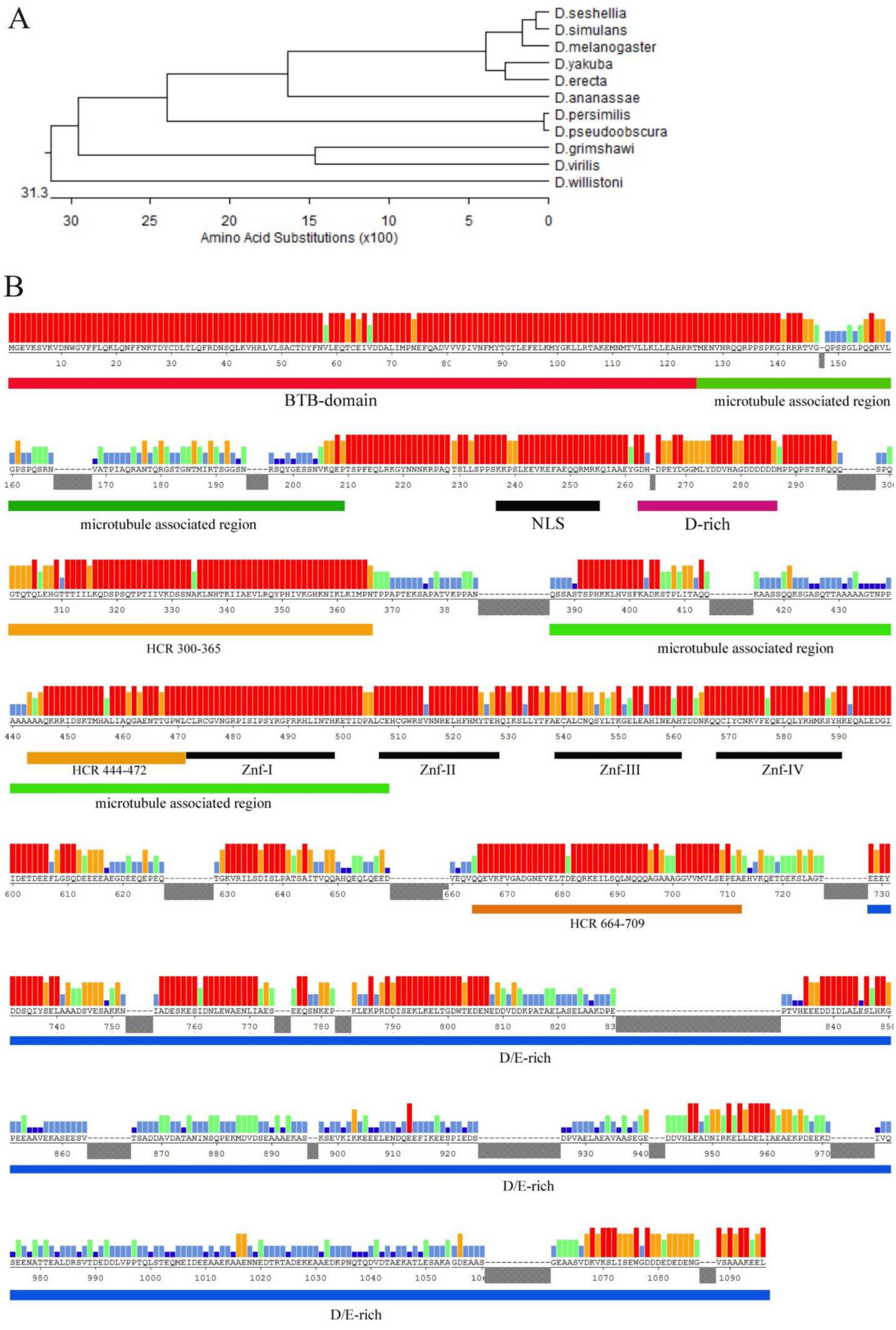
A
D.seshellia
D.simulans
D.melanogaster
D.yakuba
D.erecta
D.ananassae
D.persimilis
D.pseudoobscura
D.grimshawi
D.virilis
D.willistoni
31.3
30
25
20
15
10
5
0
Amino Acid Substitutions (x100)
B
BTB-domain
microtubule associated region
microtubule associated region
NLS
D-rich
HCR 300-365
microtubule associated region
HCR 444-472
Znf-I
Znf-II
Znf-III
Znf-IV
microtubule associated region
HCR 664-709
D/E-rich
D/E-rich
D/E-rich

Figure S1. Alignment of the CP190 proteins among *Drosophila* species. (A) Phylogenetic tree of the CP190 protein in different *Drosophila* species for which the homology of the protein was assessed. The length of each pair of branches represents the distance between sequence pairs, while the units at the bottom of the tree indicate the number of substitution events. Below the tree is a scale indicating the number of "Amino Acid Substitutions" per 100 residues for the protein sequences. (B) A schematic representation of the homology between the CP190 proteins from different *Drosophila* species generated based on multiple sequence alignment. Levels of homology for particular amino acid residues indicate the above consensus sequences as colored rectangles: red – 100%, orange -80%, green – 60%, blue -20%, and dark blue – no homology. The symbol "- -" underlined in grey indicates amino acid regions that did not fit in the consensus sequence. Under the numbers indicating the position of amino acid residues, the BTB domain is underlined in red; microtubule associated regions are underlined in green, the D-rich region by magenta, NLS (nuclear localization signal) and Zn-f (Zinc fingers) domains in black, and the D/E-rich region by blue. Highly conserved regions (HCR) identified in this work are underlined in orange, with their borders in amino acids indicated.

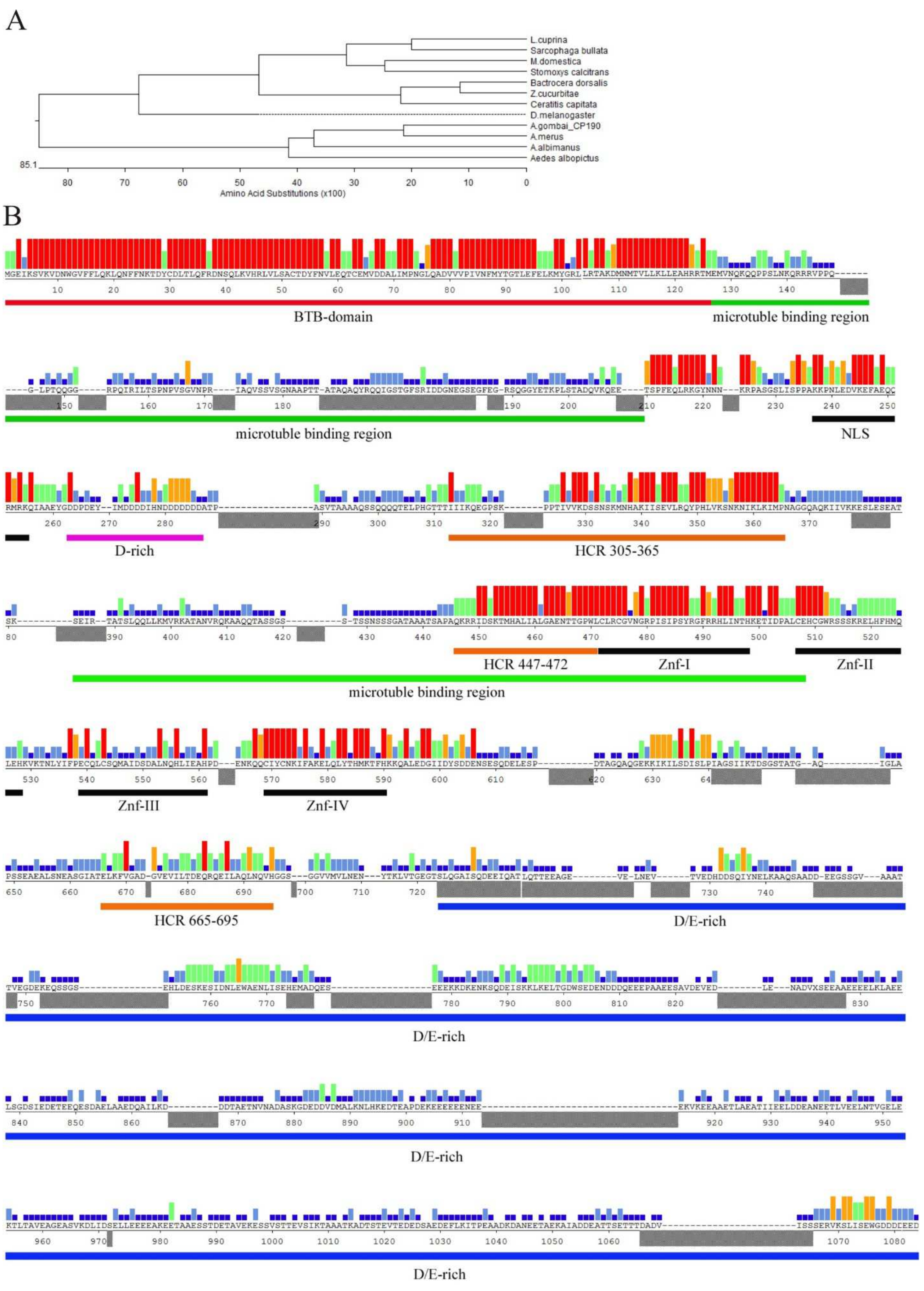

Figure S2. Alignment of the CP190 protein among species of Diptera. (A) Phylogenetic tree of the CP190 protein in different Dipteran species for which the homology of protein sequences was assessed. (B)

A schematic representation of homology between the CP190 proteins from different Dipteran species generated based on multiple sequence alignment. All designations are as in Fig. S1.

K BD /AD

1 BD-CP190(1-166)/AD

2 BD-CP190(1-220)/AD

3 BD-CP190(1-245)/AD

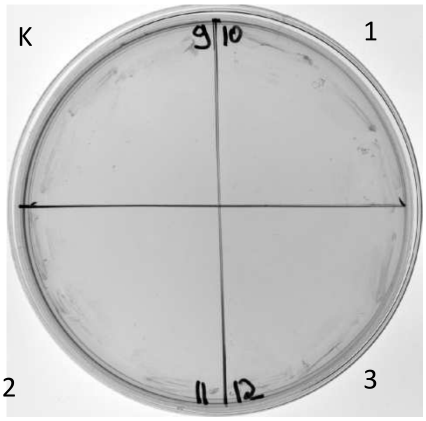

4 BD-CP190(1-293)/AD

5 BD-CP190(1-293(Δ217-245))/AD

6 BD-CP190(1-293(Δ190-210))/AD

7 BD-CP190(309-470)/AD

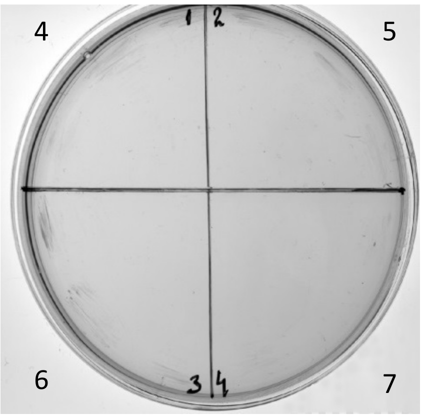

8 BD-CP190(309-390)/AD

9 BD-CP190(309-440)/AD

10 BD-CP190(1-166)/AD-ZIPIC

11 BD-CP190(1-220)/AD-ZIPIC

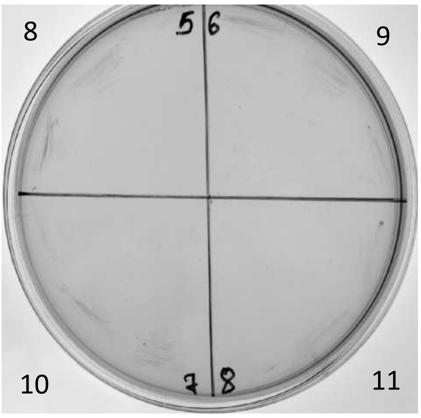

12 BD-CP190(1-245)/AD-ZIPIC

13 BD-CP190(1-293)/AD-ZIPIC

14 BD-CP190(1-293(Δ217-245))/AD-ZIPIC

15 BD-CP190(1-293(Δ190-210))/AD-ZIPIC

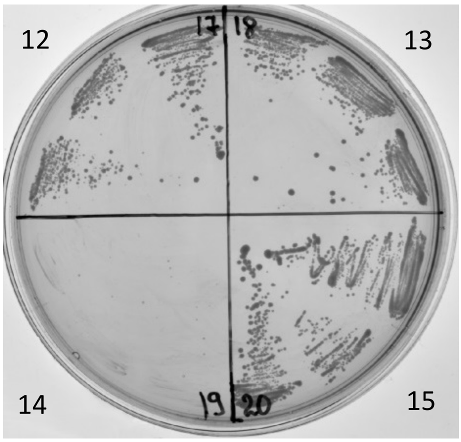

16 BD-CP190(309-470)/AD-ZIPIC

17 BD-CP190(309-390)/AD-ZIPIC

18 BD-CP190(309-440)/AD-ZIPIC

19 BD-CP190(1-166)/AD-Su(Hw)

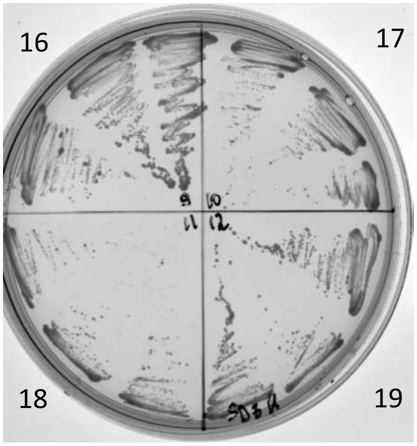

20 BD-CP190(1-293(Δ190-210))/AD-Su(Hw)

21 BD-CP190(1-293(Δ217-245))/AD-Su(Hw)

22 BD-CP190(309-470)/AD-Su(Hw)

23 BD-CP190(1-293)/AD-Su(Hw)

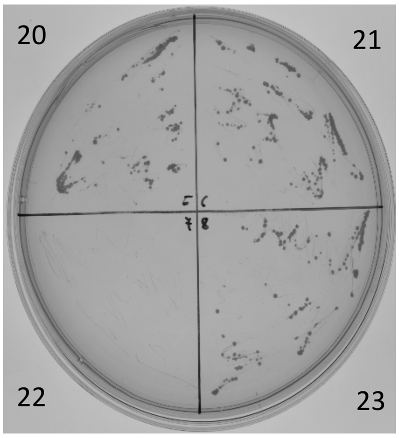

<table>
<tr>
<td>24 BD-CP190(1-245)/AD-Su(Hw)
25 BD-CP190(309-390)/AD-Su(Hw)
26 BD-CP190(1-220)/AD-Su(Hw)
27 BD-CP190(309-440)/AD-Su(Hw)
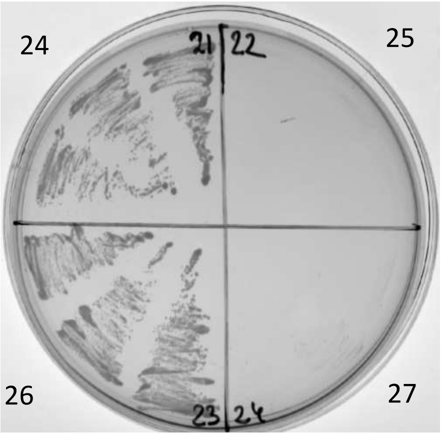</td>
<td>28 BD-CP190(1-220)/AD-M1BP
29 BD-CP190(1-220)/AD-Opbp
30 BD-CP190(1-245)/AD-M1BP
31 BD-CP190(1-245)/AD-Opbp
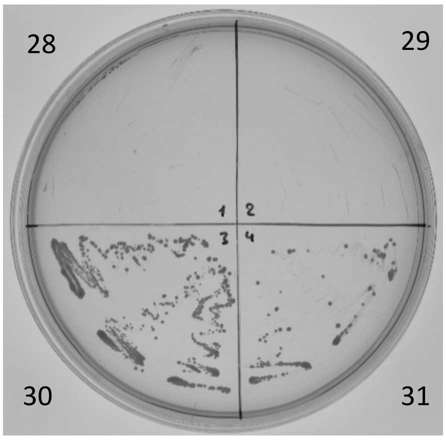</td>
<td>32 BD-CP190(1-293)/AD-M1BP
33 BD-CP190(1-293)/AD-Opbp
34 BD-CP190(1-293(Δ190-210))/AD-M1BP
35 BD-CP190(1-293(Δ190-210))/AD-Opbp
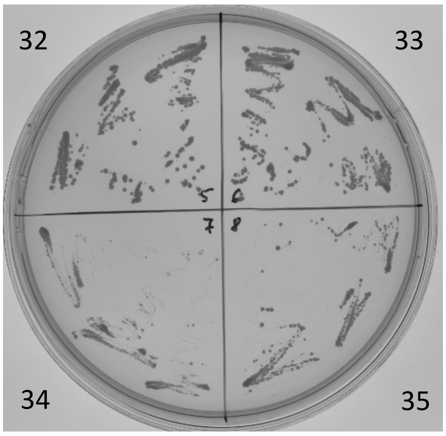</td>
</tr>
<tr>
<td>36 BD-CP190(1-293(Δ217-245))/AD-M1BP
37 BD-CP190(1-293(Δ217-245))/AD-Opbp
38 BD-CP190(309-470)/AD-M1BP
39 BD-CP190(309-470)/AD-Opbp
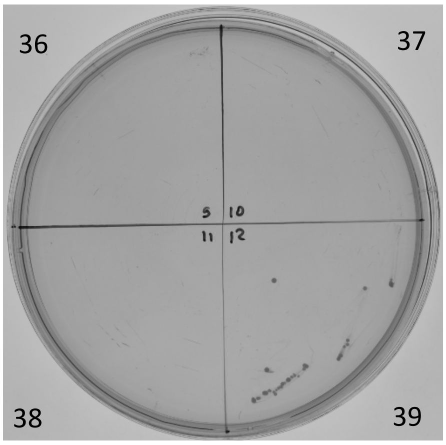</td>
<td>40 BD-CP190(309-390)/AD-M1BP
41 BD-CP190(309-390)/AD-Opbp
42 BD-CP190(309-440)/AD-M1BP
43 BD-CP190(309-440)/AD-Opbp
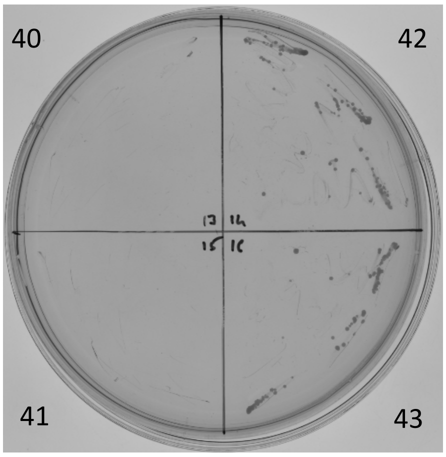</td>
<td>44 BD-CP190(1-166)/AD- CP190(1-166)
45 BD-CP190(1-166)/AD-Opbp
46 BD-CP190(1-166)/AD-M1BP
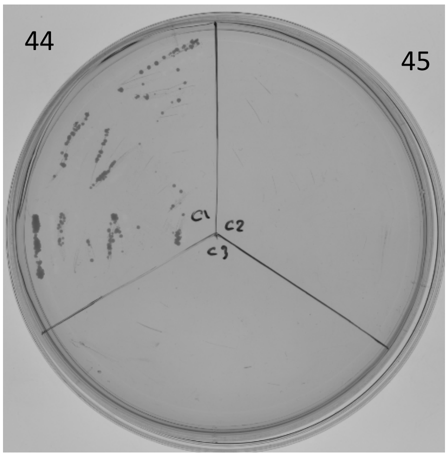
46</td>
</tr>
</table>

Figure S3. Mapping of CP190 domains interacting with Opbp, M1BP, and ZIPIC proteins in the Y2H assay. Variants of CP190 were fused to the GAL4 DNA-binding domain (BD) and tested for interaction with the C2H2 proteins fused to the GAL4 activating domain (AD).

Growth assay plates without tryptophan, leucine, histidine, and adenine are shown (yeasts are unable to grow on this medium in the absence of interaction).

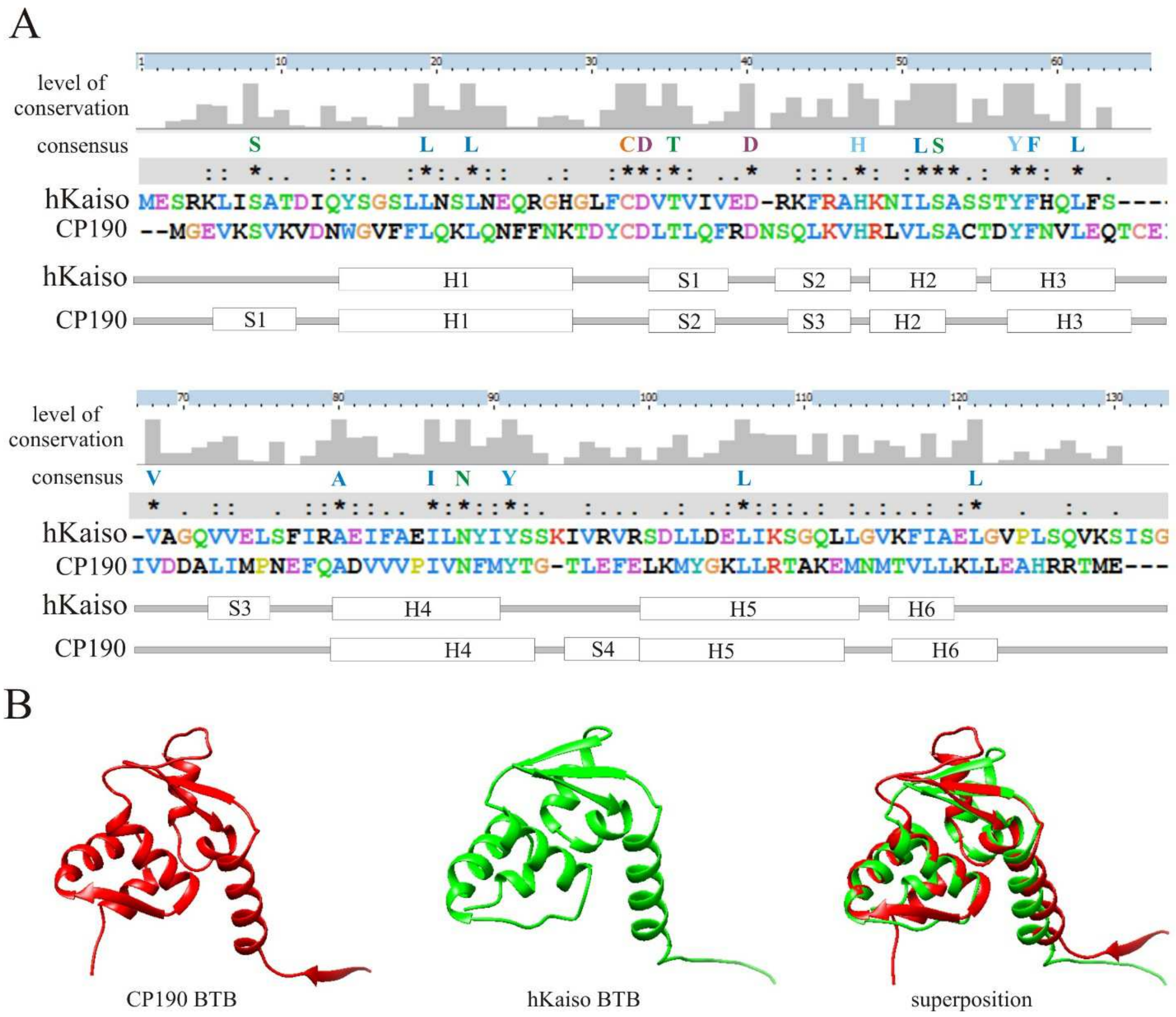

Figure S4. The CP190 BTB domain homology to the BTB domain of hKaiso transcription factor. (A) CP190 BTB protein sequence alignment to the hKaiso BTB domain. Consensus amino acid residues are shown above the alignment along with level conservation scores (grey). The “*” (asterisks) indicate positions that have a single, fully conserved residue; “:” (colon) indicates conservation between groups of strongly similar properties roughly equivalent to scoring > 0.5; “.” (dot) indicates conservation between groups of weakly similar properties roughly equivalent to scoring ≤ 0.5 and > 0. Residue letter colors correspond to the Clustal X color codes. Positions of α-helices (H) and β-strands (S) are indicated according to data from the crystal structures of several BTB domains. (B) Alphafold prediction of ribbon representation for the BTB domain of CP190 in red (left) and hKaiso in green (center). Structural superposition of CP190 and hKaiso BTB domains (right).

A

	Su(Hw)	Pita	dCTCF	BTB-CP190	BTB-hK	pGAD424
BTB-CP190	+	+	+	+	-	-
BTB-hK	-	-	-	-	+	-
pGBT9	-	-	-	-	-	ND

B

A1 BD-BTB-CP190/AD-BTB-hK

A2 BD-BTB-CP190/AD-BTB-CP190

A3 BD-BTB-CP190/AD-dCTCF

A4 BD-BTB-CP190/AD-Pita

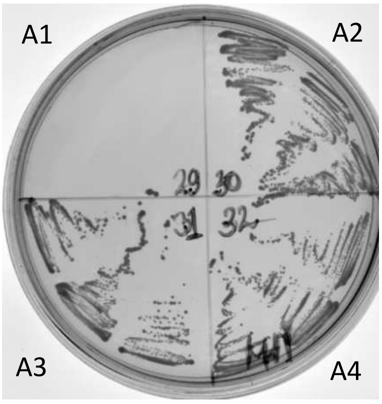

A5 BD-BTB-hK/AD-Su(Hw)

A6 BD-BTB-CP190/AD-Su(hw)

A7 BD-BTB-hK/AD-BTB-CP190

A8 BD-BTB-hK/AD-BTB-hK

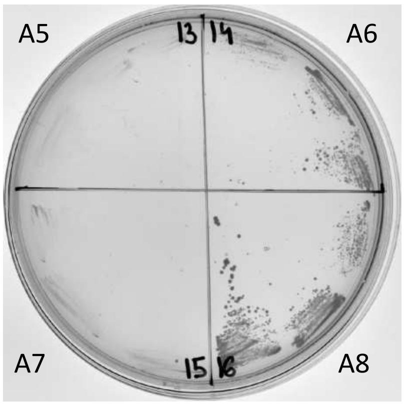

A9 BD-BTB-hK/AD-Pita

A10 BD-BTB-hK/AD-dCTCF

A11 BD/AD-Su(Hw)

A12 BD/AD-Pita

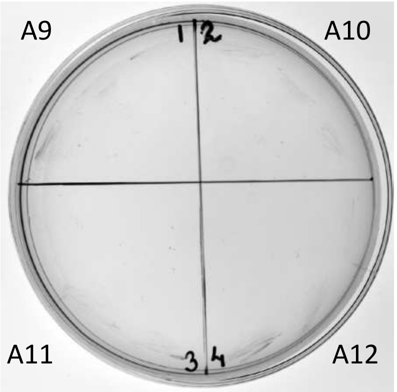

A13 BD/AD-dCTCF

A14 BD/AD-BTB-CP190

A15 BD/AD-BTB-hK

A16 BD-BTB-hK/AD

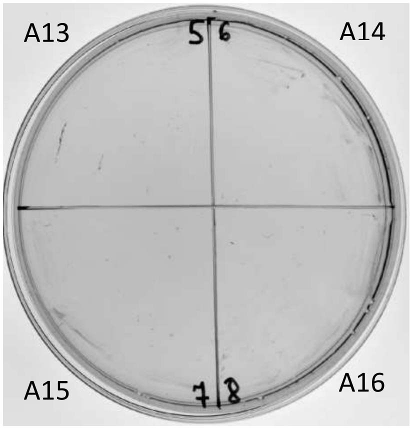

A17 BD-BTB-CP190/AD

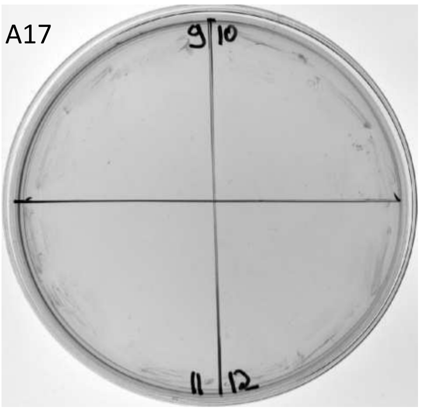

Figure S5. Comparing the BTB domains of CP190 and Kaiso for interaction with Su(Hw), Pita, and dCTCF in the Y2H assay. (A)The BTBs were fused to the GAL4 DNA-binding domain (BD) or the GAL4

activating domain and assayed for self-interaction or interaction with the C2H2 proteins fused to the GAL4 activating domain (AD). The results are summarized in columns, with “+” and “−” signs referring to the presence and absence of an interaction, respectively. (B) . Growth assay plates without tryptophan, leucine, histidine, and adenine are shown.

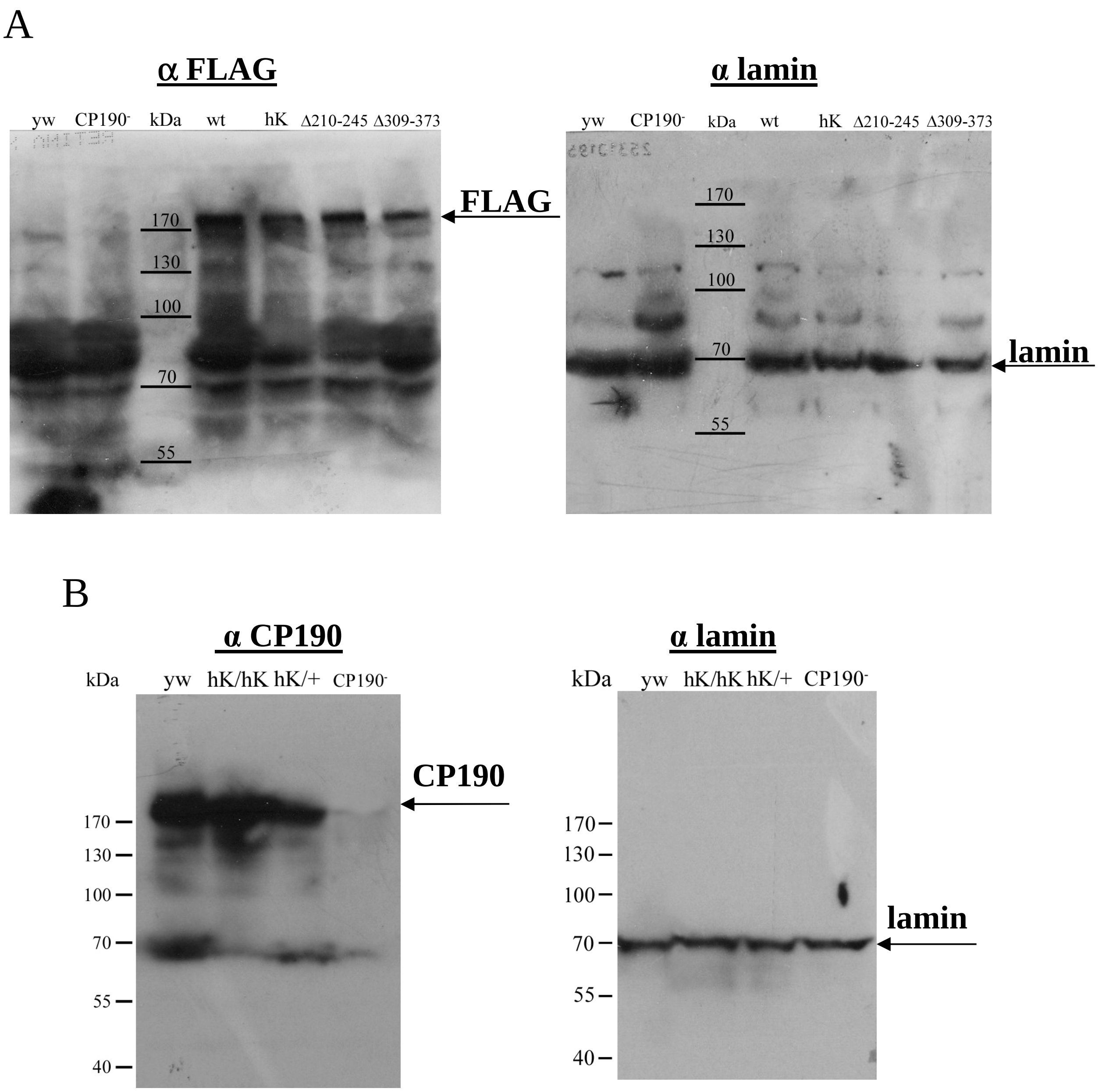

Figure S6. Immunoblot analysis (10 % SDS PAGE) of protein extracts from the lines expressing CP190 variants. (A) The protein extract was prepared from third-instar larvae of the transgene lines expressing different heterozygote variants of the CP190 protein: $CP190^{WT}$-F (WT), $CP190^{hK}$–F (hK) $CP190^{\Delta 210\text{-}245}$–F (Δ210-245), $CP190^{\Delta 309\text{-}373}$–F (Δ309-373). $Cp190^{2}/Cp190^{3}$ ($Cp190^{-}$) and $y^{1}w^{1118}$ (yw) lines were used as negative controls. The membrane was sequentially stained with anti-FLAG (α FLAG) antibodies to detect the level of CP190 expression and anti-lamin (α lamin) antibodies as a loading control. Molecular weights in kDa are marked on the right. (B) The protein extract was prepared from adult two-day-old males of the lines expressing homozygote or heterozygote $CP190^{hK}$–F (hK) variants to compare to the wild type CP190 expression level in the $y^{1}w^{1118}$ (yw) line. The $Cp190^{-}$ line was used as a negative control. The membrane was sequentially stained with anti-CP190 (α CP190) and anti-lamin (α lamin, loading control) antibodies.

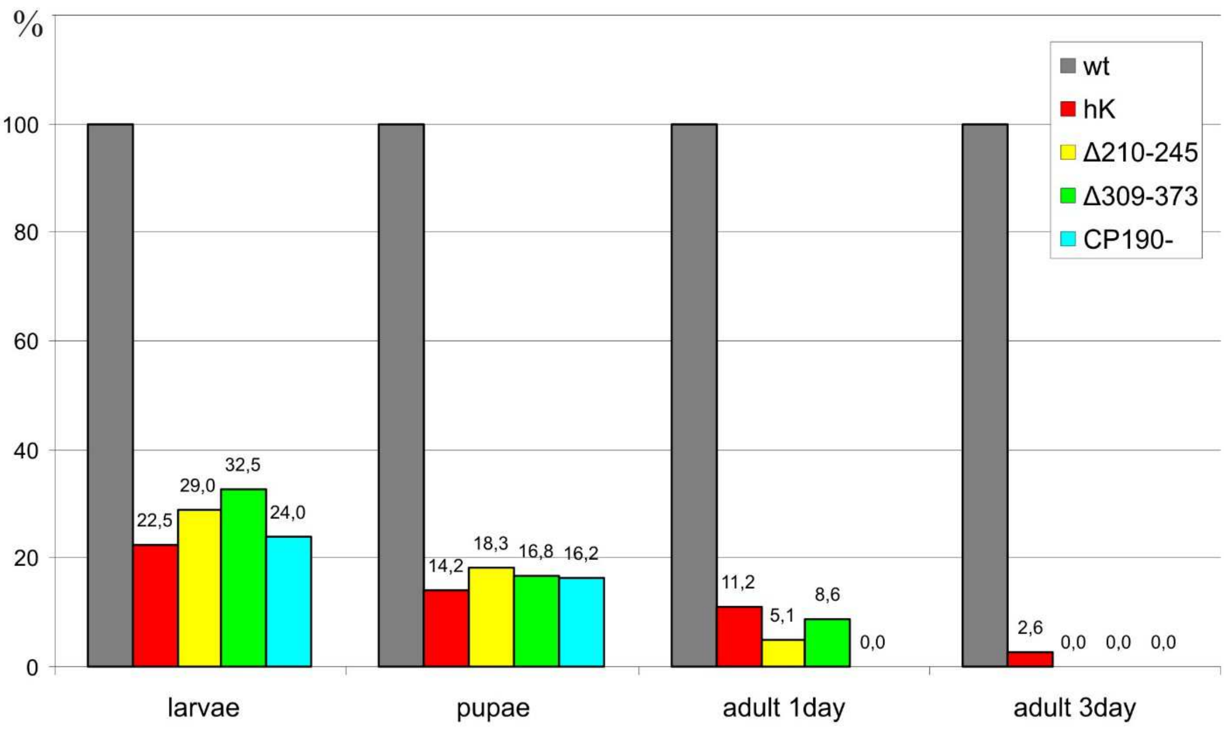

Figure S7. Viability of flies expressing different variants of CP190. Viability of flies was calculated for the crosses ♂ *Ubi:CP190*/Ubi:CP190*; Cp190*^{2}*/TM3-GFP (TM3, P{w[+mC]=GAL4-Kr.C}DC2, P{w[+mC]=UAS-GFP.S65T}DC10, Sb[1]) x* ♀ *+/+; Cp190*^{3}*/TM3-GFP*. Viability was calculated as percent ratios (numbers above the bars) of *Ubi:CP190*/+; Cp190*^{2}*/Cp190*^{3} flies to *Ubi:CP190*^{WT}*/+; Cp190*^{2}*/Cp190*^{3} flies. Ubi:CP190* is any transgene expressing one of the tested CP190 variants: Ubi:CP190^{hK}(hK), Ubi: CP190$^{\Delta 210\text{-}245}$(Δ210-245), or Ubi: CP190$^{\Delta 308\text{-}373}$(Δ308-373). The *Cp190*^{2}*/Cp190*^{3} (Cp190^{-}) line was used as a control. For statistical analysis, 200 *Cp190*^{2}*/Cp190*^{3} larvae from each cross were collected, and their viability was monitored at different stages of development: larvae, pupae, and adults on the first and third days after hatching.

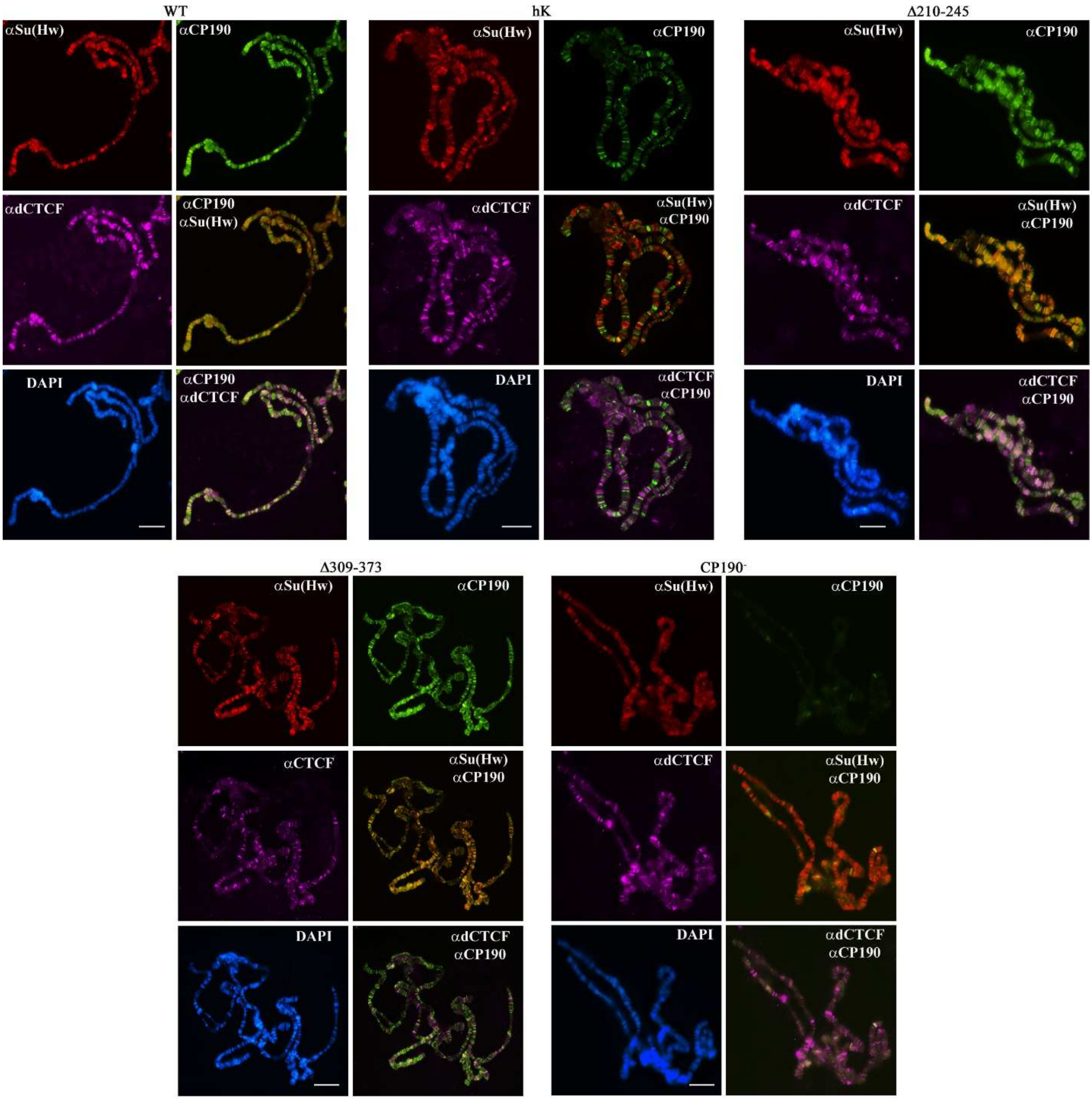

Figure S8. Testing the ability of CP190 variants to bind to Su(Hw) or dCTCF sites on the polytene chromosomes. Cytological localization of CP190, dCTCF and Su(Hw) proteins on the polytene chromosomes of the *Ubi:CP190*/CyO; Cp190*^{2}*/Cp190*^{3} lines, where *Ubi:CP190** is either *Ubi:CP190*^{WT}(WT), *Ubi:CP190*^{hK}(hK), *Ubi: CP190*$^{\Delta 210\text{-}245}$(Δ210-245) or *Ubi: CP190*$^{\Delta 309\text{-}373}$(Δ309-373). CP190^{-} represents the *Cp190*^{2}*/Cp190*^{3} mutant line. The panels show the immunostaining using rabbit anti-Su(Hw) (red), mouse anti-dCTCF (magenta), and rat anti-CP190 antibodies (green). DAPI staining of polytene chromosome squashes is shown in blue. Scale bars, 20 μm.

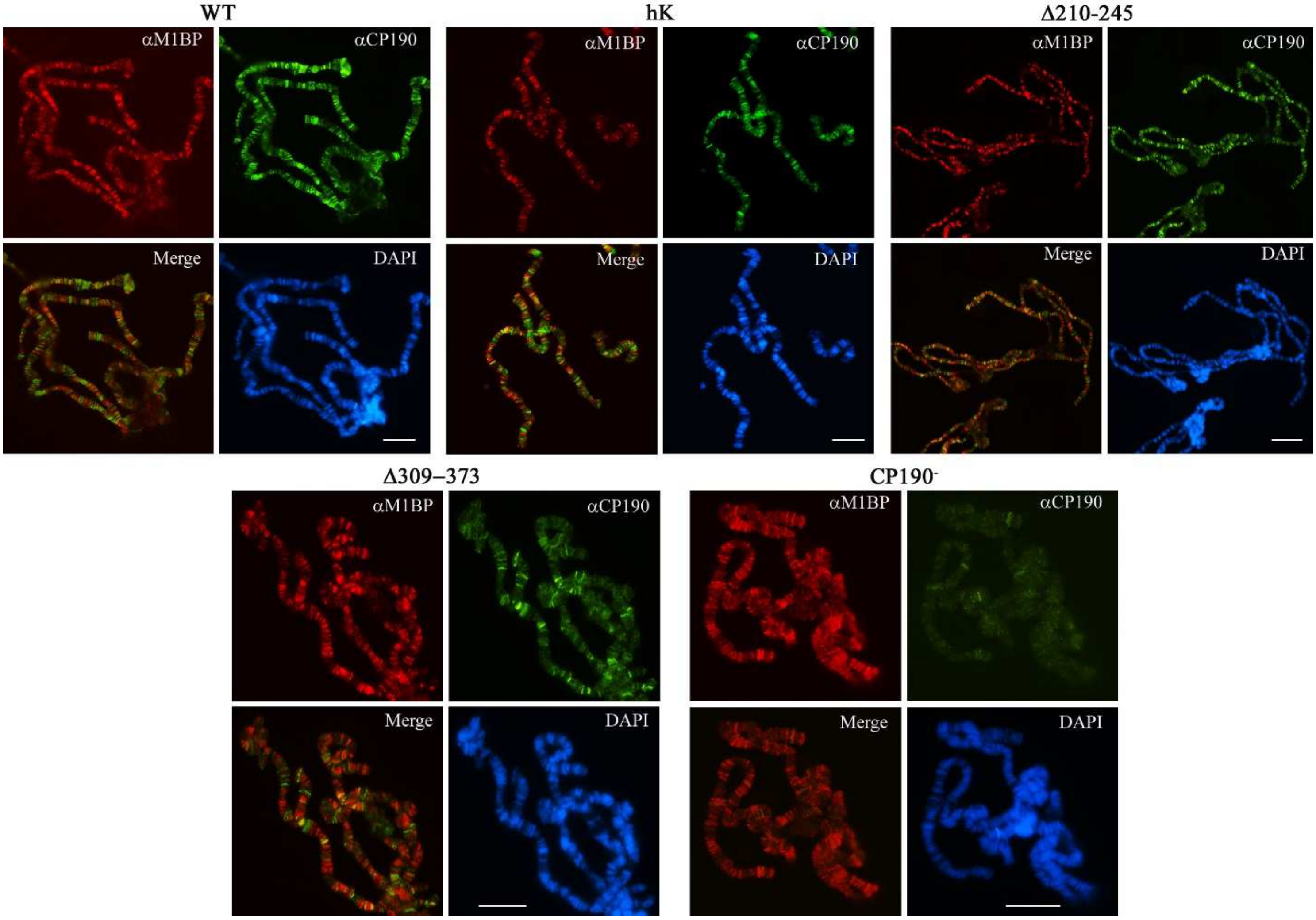

Figure S9. Testing the ability of CP190 variants to bind to M1BP sites on the polytene chromosomes. Cytological localization of CP190 and M1BP proteins on the polytene chromosomes. The panels show the immunostaining using rat anti-CP190 (green) and rabbit anti-M1BP (red) antibodies and DAPI (blue). All designations are as in Fig. S7. Scale bars, 20 μm.

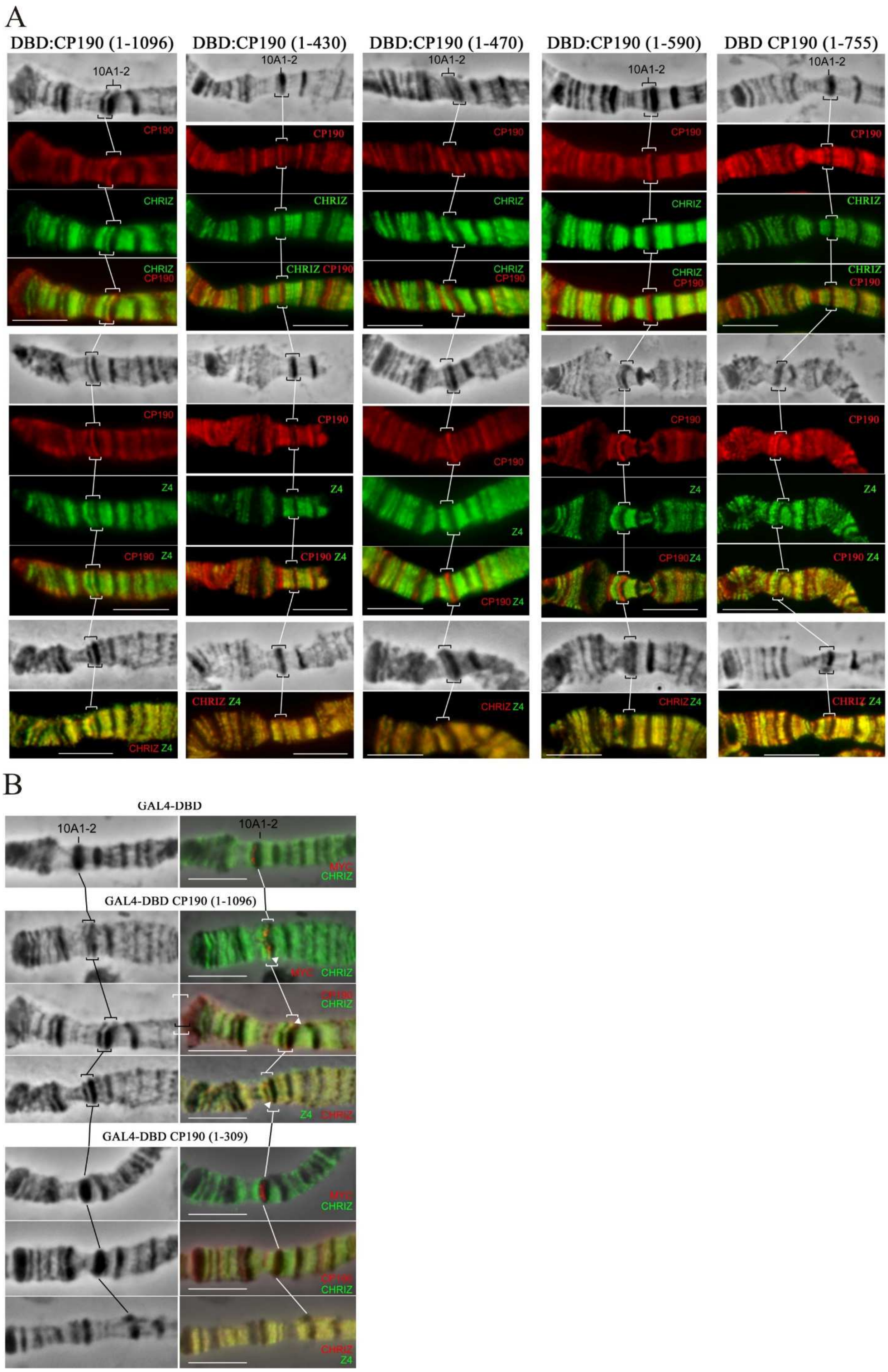
A
DBD:CP190 (1-1096)
DBD:CP190 (1-430)
DBD:CP190 (1-470)
DBD:CP190 (1-590)
DBD CP190 (1-755)
10A1-2
CP190
CHRIZ
Z4
B
GAL4-DBD
GAL4-DBD CP190 (1-1096)
GAL4-DBD CP190 (1-309)
MYC
CHRIZ
CP190
Z4

Figure S10. Mapping of the region in CP190 required to organize interbands on a polytene chromosome model.

(A) Targeting the GAL4 DNA-binding region fused with CP190 derivatives and the *myc* epitope to the 16 GAL4 binding sites in the 10A1-2 disc. The CP190 derivatives represent different truncated variants of the protein: GAL4-DBD:CP190(1-430), GAL4-DBD:CP190(1-470), GAL4-DBD:CP190(1-590) and GAL4-DBD:CP190(1-755). As positive control full length CP190 (GAL4-DBD:CP190(1-1096)) was used. There is a panel demonstrating the polytene chromosomes in phase contrast for each independent squash. Under the phase contrast panel, there are panels with an overlay of phase contrast and immunostaining with antibodies against CP190 (red), Chriz (green), or Z4 (green), and they overlay in different combinations. Black or white square brackets indicate model 10A1-2 band on different polytene chromosome preparations. All variants induce the formation of the interband in the 10A1-2 band. (B) Targeting the GAL4 DNA-binding (GAL-DBD) region fused with *myc* epitope, 1-1096 aa CP190 (GAL4-DBD:CP190 1-1096) or GAL4-DBD:CP190(1-309) regions fused with the GAL4 DNA-binding region and the *myc* epitope to the 16 GAL4 binding sites in the 10A1-2 disc. The left panel demonstrates the polytene chromosomes in phase contrast. The right panel is an overlay of phase contrast and immunostaining with antibodies against *myc* (red), Chriz (green), Z4 (green), and CP190 (red). Black or white square bracket lines indicate model 10A1-2 band on different polytene chromosome preparations. At the top, the recruitment of the GAL4 DNA-binding region alone (DBD) did not induce the formation of the interband in the 10A1-2 band (negative control). In the middle, the recruitment of the GAL4 DNA-binding region fused with 1-1096 aa CP190 induced the formation of the interband in the 10A1-2 band (positive control). At the bottom, the recruitment of the GAL4 DNA-binding region fused with 1-309 aa CP190 (GAL4-DBD:CP190(1-309)) did not induce the formation of the interband. Scale bars, 10 μm.

Supplementary Materials

M1BP recombinant protein purification

To generate M1BP epitope cDNA fragment coding 90-240 aa region was amplified with primers 5' ttGGATCCgagcaacgccaaggcatc 3' and 5' ttGTCGACatccgaccggacctttggc 3'. Resulting PCR fragment was digested with BamHI-SalI and cloned in to modified pET32a(+) vector (Sigma cat#69015, St. Louis, MO, USA) (with deleted thioredoxin tag) digested by BamHI and SalI in-frame with 6xHis-tag.

Protein expression and purification were performed using standard procedures. Briefly, BL21 cells were disrupted by sonication in buffer A (40 mM HEPES-KOH pH 7.7, 400 mM NaCl, 5 mM β-mercaptoethanol, 20 mM Imidazole) containing 1 mM PMSF and Calbiochem Complete Protease Inhibitor Cocktail VII (1µL/1ml). After centrifugation, lysate was applied to Ni-NTA column, and after washing, it was eluted with 300 mM Imidazole and dialyzed against an appropriate buffer.
